# Differentially Expressed MiRNAs of Goat Submandibular Glands Among Three Developmental Stages Are Involved in Immune Functions

**DOI:** 10.3389/fgene.2021.678194

**Published:** 2021-06-15

**Authors:** Aili Wang, Zhibin Ji, Rong Xuan, Xiaodong Zhao, Lei Hou, Qing Li, Yunpeng Chu, Tianle Chao, Jianmin Wang

**Affiliations:** ^1^Shandong Provincial Key Laboratory of Animal Biotechnology and Disease Control and Prevention, College of Animal Science and Veterinary Medicine, Shandong Agricultural University, Taian, China; ^2^Shandong Peninsula Engineering Research Center of Comprehensive Brine Utilization, Weifang University of Science and Technology, Shouguang, China

**Keywords:** different developmental stages, goat, submandibular gland, high-throughput sequencing, differentially expressed miRNAs

## Abstract

Submandibular glands (SMGs) are one of the primary components of salivary glands in goats. The proteins and biologically active substances secreted by the SMGs change with growth and development. Our previous studies showed that most of the differentially expressed genes in the SMGs of goats at different developmental stages are involved in immune-related signaling pathways, but the miRNA expression patterns in the same tissues are unknown. The aim of this study was to reveal the expression profile of miRNAs at three different developmental stages, detect differentially expressed miRNAs (DE miRNAs) and predict disease-related DE miRNAs. SMG tissue samples were collected from groups of 1-month-old kids, 12-month-old maiden goats and 24-month-old adult goats (three samples from each group), and high-throughout transcriptome sequencing was conducted. A total of 178, 241 and 7 DE miRNAs were discovered between 1-month-old kids and 12-month-old maiden goats, between 1-month-old kids and 24-month-old adult goats, and between 12-month-old maiden goats and 24-month-old adult goats, respectively. Among these DE miRNAs, 88 DE miRNAs with medium or high expression levels (TPM ≥50) were classified into five expression pattern clusters. Gene Ontology (GO) and Kyoto Encyclopedia of Genes and Genomes (KEGG) analyses indicated that some of the predicted target genes of the DE miRNAs in the five clusters were enriched in disease-related GO terms and pathways. MiRNA target genes in significant pathways were significantly enriched in Hepatitis B (FDR = 9.03E-10) and Pathways in cancer (FDR = 4.2E-10). Further analysis was performed with a PPI network, and 10 miRNAs were predicted to play an important role in the occurrence and prevention of diseases during the growth and development of goats.

## Introduction

The submandibular glands (SMGs) are one of the primary components of salivary glands in mammals. SMGs lie under the tongue and develop in set positions within the mouth (Tucker, 2007). In goats, the SMGs are mixed gland containing both serous and mucous acinar cells; in addition, the SMGs are the first major salivary gland to develop in the embryo. Mucous acinar cells mainly secrete mucins, which increase the viscosity of saliva, and serous acinar cells secrete a large number of proteins, which participate in physiological and biochemical reactions during growth and development (Kameyama et al., 2020).

The SMGs change with growth and development, and these changes may influence the secretion process and result in secretory granules with irregular shapes (D'Avola et al., 2006). Studies have shown that the proteins and biologically active substances secreted by SMGs change with age. The levels of hormones and epidermal growth factor (EGF) in rat SMGs gradually increase with age from 4 to 8 weeks (Hiramatsu et al., 1994). The level of carbonic anhydrase VI (CA VI) expression in ovine SMGs between days 7 and 59 is dramatically increased (~600-fold), as determined by radioimmunoassay (Penschow et al., 1997). In the SMGs of postnatal rats, substance P increases in surges during the first 8 weeks of life (Ekström et al., 1994). In addition to the types of substances, their proportions in the SMGs change with growth and development. Kameyama et al. found that both types of mucins secreted in the mouse SMGs and the proportions of the mucin glycan species expressed throughout life change during the aging process (Kameyama et al., 2020).

MiRNAs, a class of non-coding RNA molecules with lengths of ~22 nucleotides (nt), exert a negative regulatory effect on gene expression through the inhibition and degradation of target mRNAs by binding to target mRNAs through sequence-specific base pairing after gene transcription (Hudder and Novak, 2008). MiRNAs play an important role in cellular metabolism, growth, proliferation and the immune response. In cells, protein expression levels are regulated and further influenced by gene expression; some physiological and biochemical indicators of biological functions are regulated in this way. Previous studies have shown that miRNA expression exhibits certain tissue specificity, and miRNA expression is different in different developmental stages. Conserved and novel miRNAs were identified in dairy goat mammary glands (Ji et al., 2012, 2017), goat endometrium during embryo implantation (Song et al., 2015), testes (Wu et al., 2014), skin, and hair follicles by Solexa sequencing (Yuan et al., 2013). Specific changes in urinary miRNA expression in male goats during the breeding season may be related to their high testosterone levels during breeding (Longpre et al., 2014). Serum miRNA expression profiles are different in Chongming white goats pre- and post-weaning (Liao et al., 2019).

MiRNAs 103 and 107 are potent regulators of glucocorticoid receptor (GR) function in hypoxia (Yang et al., 2020). Goat skeletal muscle satellite cell proliferation can be regulated by miR-101a (Li et al., 2015). Many miRNAs were found in exosomes and milk fat globules of human and cow milk; these miRNAs include miRNA-148a, which attenuates the expression of DNA methyltransferase 1, and miRNA-125b, which targets p53, which is considered the guardian of the genome (Melnik and Schmitz, 2017). In the goat mammary gland, fat metabolism is suppressed by miR-181b, which regulates multiple genes in the Hippo pathway and targets insulin receptor substrate 2 (IRS2) (Chen Z. et al., 2016). In goat mammary epithelial cells, fat metabolism is suppressed by the miR130b-mediated regulation of *PPAR*γ coactivator-1α (Chen et al., 2015). Fatty acid metabolism is regulated by miR-148a, which cooperates with miR-17-5p, repressing *PPARGC1A* and *PPARA* in goat mammary epithelial cells (GMECs) (Chen et al., 2017). MiR-34c promotes apoptosis in goat male germline stem-cells (mGSCs) in a p53-dependent manner and reduces their proliferation (Li et al., 2013). In dairy goats, spermatogonial stem cell proliferation is regulated by Sirt1, which is regulated by miR-204 (Niu et al., 2016).

MiRNAs play a significant role in immune responses. MiRNAs are critical for lymphocyte development and differentiation, and a deficiency in miRNAs also affects T and B cell development (Xiao and Rajewsky, 2009). miRNA-deficient mice display severe immune deficiency, particularly impaired B cell and T cell responses (Turner and Vigorito, 2008; Roy and Sen, 2011). The miR-146 family was predicted to regulate the gene expression of TNF receptor-associated factor 6 and IL-1 receptor-associated kinase 1 by binding to sequences in the 3′ UTRs of these genes. UTRs inhibit the expression of associated reporter genes and Toll-like and cytokine receptors (Taganov et al., 2006). MiRNAs induced by proinflammatory stimuli, such as miR-146, miR-155, and miR-21, have been of great interest in investigations of inflammatory and immune responses. In the immune system, miR-155, which is predicted to regulate a broad range of inflammatory mediators, has emerged as a central regulator (Roy and Sen, 2011). Increased expression of miR-221 and miR-222, which regulate brahma-related gene 1 (*Brg1*), correlates with increased immunoparalysis and organ damage, and specific miRNAs may serve as biomarkers of immunoparalysis (Seeley et al., 2018). The DE miRNAs miR-146, miR-155, miR-181a, miR-223, and miR-150 were all detected in goat milk. miR-223 was found at higher levels in goat colostrum than in human colostrum, while the expression levels of miR-146 and miR-155 were absolutely opposite, and these miRNAs are significantly more highly expressed in colostrum than in mature milk (Na et al., 2015). In dairy cows with *Staphylococcus aureus*-induced mastitis, immune cytokines are regulated by miR-145, which targets and regulates *FSCN1*, and downregulating miR-145 and upregulating *FSCN1* expression promotes mammary epithelial cell proliferation and accelerates the recovery of damaged tissues (Chen Z. et al., 2019). However, studies on the effects of miRNAs of SMGs were mainly focused on humans, mice and other animal models, the miRNA of goat SMGs is rarely reported.

In our previous study, the mRNA expression in the SMGs of goats were distinctly different at different developmental stages, and immune-related genes/pathways were identified (Wang et al., 2020). Numerous mRNAs, including mRNA encoding 8 antimicrobial peptides, were highly expressed in the SMGs of 1-month-old kids and hardly expressed in 12-month-old maiden goats and 24-month-old adult goats, while the immunoglobulin expression pattern was the opposite, with low expression in the SMGs of 1-month-old kids and high expression in 12-month-old maiden goats and 24-month-old adult goats. Since miRNA data of the SMGs of goats at different developmental stages are lacking, it is difficult for us to study the relationship between miRNA and gene expression in this tissue. Therefore, in this study, the miRNA profiles of SMG samples from 1-month-old kid goats, 12-month-old maiden goats, and 24-month-old adult goats were described, and DE miRNAs were investigated. These studies are helpful for exploring the potential roles of miRNAs during goat development. The results expanded the repertoire of known goat miRNAs and may help to better understand the function and underlying mechanisms of goat SMGs.

## Materials and Methods

### Ethics Statement

All the animal experiments, which were conducted in accordance with the “Guidelines for Experimental Animals” of the Ministry of Science and Technology (Beijing, China), were approved by the Committee on the Management and Use of Laboratory Animals of Shandong Agricultural University (Permit Number: SDAUA-2018-052). Every effort was made to minimize pain.

### Experimental Sample Collection

The experimental animals were obtained from Shandong Fengxiang Animal Husbandry and Seed Industry Technology Co., Ltd. (Laiwu District, Jinan, Shandong Province, China). In this experiment, goats were divided into three different groups based on their developmental stages: the 1-month-old kids (group A), the 12-month-old maiden goats (group B) and the 24-month-old adult goats (group C). Three healthy female goats (Lubo goats) were included in each group (nine goats in total). All the goats were raised under the same conditions with natural lighting and free access to food. Before slaughter, all the goats were fasted from food for 24 h and fasted from liquids for 2 h, and then, xylazine hydrochloride (Huamu Animal Health Products Co., Ltd., China) was injected intramuscularly to induce general anesthesia. After slaughter and exsanguination, the SMGs were immediately collected surgically, placed in liquid nitrogen and then placed in a −80°C freezer for long-term storage. To distinguish the samples, the submandibular salivary gland samples from 1-month-old kids were labeled A1-s, A2-s and A3-s; the samples from 12-month-old maiden goats were labeled B3-s, B4-s and B5-s; and the samples from 24-month-old adult goats were labeled C2-s, C3-s and C5-s.

### RNA Extraction, Library Construction, and Sequencing

Total RNA was extracted from SMGs tissues using TRIzol (Invitrogen, Carlsbad, CA, USA). The RNA molecules in a size range of 18–30 nt were then enriched by polyacrylamide gel electrophoresis (PAGE). The 3′ adapters were added, and the 36–44 nt RNAs were enriched. The 5′ adapters were then also ligated to the RNAs. The ligation products were reverse transcribed by PCR amplification, and the 140–160 bp PCR products were enriched to generate a cDNA library and sequenced using Illumina HiSeqTM 2500 by Gene Denovo Biotechnology Co. (Guangzhou, China).

### Sequencing Data Quality Control

To obtain clean reads, raw reads were further filtered with FastQC (Cock et al., 2010) according to the following rules: remove low-quality reads containing more than one low-quality (Q-value ≤ 20) base or containing unknown nucleotides (N); remove reads without 3′ adapters; remove reads containing 5′ adapters; remove reads containing 3′ and 5′ adapters but no small RNA fragment between them; remove reads containing polyA in small RNA fragments (More than 70% of the bases in a single read are A); and remove reads shorter than 18 nt (not including adapters).

### Sequencing Data Alignment and miRNA Identification

With Bowtie (version 1.1.2) (Langmead and Salzberg, 2012), we aligned our clean reads with small RNAs in the GenBank database (Release 209.0) and Rfam database (11.0) to identify and remove rRNAs, scRNAs, snoRNAs, snRNAs, and tRNAs. We also mapped our clean reads with the goat reference genome (*Capra hircus* ARS1), and the reads that mapped to exons, introns and repeat sequences were removed. To identify known miRNAs, we mapped the clean reads with the miRBase database (Release 22). All of the unmapped reads were used for novel miRNA identification according to their genome positions and hairpin structures predicted by Mireap_v0.2 software (Chen L. et al., 2019). The parameters of Mireap_v0.2 were established as follows: minimal miRNA sequence length is 18 nt, maximal miRNA sequence length is 26 nt, minimal miRNA reference sequence length is 20 nt, maximal miRNA reference sequence length is 24 nt, minimal depth of Drosha/Dicer cutting site is 3, maximal copy number of miRNAs on reference is 20, maximal free energy allowed for a miRNA precursor is 18 kcal/mol, maximal space between miRNA and miRNA^*^ is 35 nt, minimal space between miRNA and miRNA^*^ is 14 nt, maximal bulge between miRNA and miRNA^*^ is 4 nt, maximal asymmetry of miRNA/miRNA^*^ duplex is 5 nt, and flank sequence length of miRNA precursor is 10 nt. To distinguish known miRNAs in goat and other species, the 5 p and 3 p in the names of miRNA were denoted by “x” and “y,” respectively.

### MiRNA Expression Profile and PCA Analysis

For both known miRNAs and novel miRNAs, the miRNA expression level was calculated and normalized by transcripts per million (TPM). The formula was as follows: TPM = Actual miRNA counts/Total counts of clean tags^*^106. The principal component analysis was performed using TPM of all miRNAs by R package (http://www.r-project.org/).

### Differentially Expressed miRNA Analysis

DE miRNAs across different developmental stages were identified with DESeq2 (version 1.10.1) (Love et al., 2014). A fold change ≥2 and a false discovery rate value (FDR) <0.05 were set as cutoffs to determine significant differential expression. Then, we performed target gene prediction for all the DE miRNAs with RNAhybrid (v2.1.2) (Krüger and Rehmsmeier, 2006), Miranda (v3.3a) (Enright et al., 2003), and TargetScan (v7.0) (Sethupathy et al., 2006). The intersection results from three software programs were chosen as predicted miRNA target genes. For RNAhybrid, threshold were as follows: forces structures to have a helix from position 2–8 with respect to the query; the number of hits per target is 1; maximal bulge loop size is 3 nt; maximal internal loop size (per side) is 3 nt; the cutoff *P*-value is 0.05; the cutoff energy is −10 kcal/mol; and maximal query length is 24 nt. For Miranda, the set score threshold is 140; the set energy threshold is −10 kcal/mol; demand strict 5′ seed pairing; gap-open penalty is−4.0; and the gap-extend penalty is−9.0. For TargetScan, 2–8-nt sequences starting from 5′ small RNA were chosen as seed sequences to predict the 3′ UTR of the transcripts. After removing the miRNAs with low expression levels (TPM <1), the DE miRNA expression scale of nine groups of samples was standardized by *Z*-Score.

Family analysis was conducted based on sequence similarity and the conservation of miRNAs among different species. According to the average TPM values of three samples in each developmental stage, the miRNAs were divided into four groups: extremely high-expression group (TPM ≥ 5,000), high-expression group (5,000 >TPM ≥ 500), medium-expression group (500 > TPM ≥ 50), and low-expression group (50 > TPM ≥1).

### Expression Pattern Analysis of DE miRNAs

To further understand the expression pattern of the DE miRNAs with TPM ≥ 50 in the SMGs of goats at three different developmental stages, expression pattern analysis of the DE miRNAs was performed with TCseq software (1.14.0) (http://bioconductor.org/packages/release/bioc/html/TCseq.html) (Xuan et al., 2020) using the c-means method with the parameter dist = “Euclidean,” algo = “cm,” *k* = 5. Line graphs were drawn with the expression levels of the DE miRNAs of the goats in three different developmental stages.

### Target Gene Prediction and Functional Enrichment Analysis

Schober et al. reported that−1.0 < Pearson correlation coefficient < -0.90 was strong or high correlation, and the correlation coefficient of 0.65 could either be interpreted as a “good” or “moderate” correlation (Schober et al., 2018). To generate a comprehensive description of the biological characteristics of the DE miRNAs, we mapped all of the candidate target genes to the Gene Ontology (GO) database (http://www.geneontology.org/) and Kyoto Encyclopedia of Genes and Genomes (KEGG) (https://www.genome.jp/kegg/) using the R package clusterProfiler (Alexa and Rahnenführer, 2009; Yu et al., 2012) with the screening standard FDR, calibrated after the *p*-value. The terms and pathways with FDR <0.05 were considered significantly enriched pathways.

### Network Analysis

PPI network analysis of the target genes in significantly enriched pathways was performed by Metascape (http://metascape.org/) and Cytoscape 3.7.2. Ten target genes were chosen as key genes according to interactions from large to small. According to a Pearson correlation coefficient < -0.7 and a *p*-value < 0.05, the top 10 core gene targets of the miRNAs above were predicted. The top 10 miRNAs were selected as core miRNAs according to the order of miRNA expression and fold change in differential expression from large to small. Then, regulatory networks of the miRNAs and potential target genes that were significantly enriched in the disease-related KEGG pathways were drawn by Cytoscape 3.7.2.

### Quantitative Real-Time PCR (qRT-PCR) Validation

To further verify the differential expression of the miRNAs in the nine goat SMG samples as determined by high-throughput sequencing, we randomly selected 20 differentially expressed miRNAs for RT-qPCR detection. The experiment was carried out using a LightCycler 480 real-time system (Roche, USA). The qRT-PCR primers used for validation are listed in Supplementary Table 1 and were designed according to the sequences of the selected DE miRNAs using Primer Premier 5.0 (Premier, Canada). qRT-PCR was performed according to the instructions of the Mir-X™ miRNA qRT-PCR TB Green™ Kit (Clontech). A reaction volume of 25 μl with 12.5 μl TB Green Advantage Premix (2 × ), 0.5 μl ROX Dye (50X), 0.5 μl miRNA-specific primer (10 μM), 0.5 μl mRQ 3′ Primer (10 μM), 2.0 μl cDNA, and 9 μl ddH_2_O was used. The reaction mixtures were incubated in a 96-well plate at 95°C for 10 s, followed by 40 cycles of 95°C for 5 s and 60°C for 20 s, then 95°C for 60 s, 55°C for 30 s and 95°C for 30 s. U6 was used as the reference gene. All the reactions were performed in triplicate, and the results were calculated using the 2^−ΔΔCt^ method (Livak and Schmittgen, 2001). Tukey's honestly significant difference (HSD) test was used to test the significance of mRNA expression, and miRNA expression histograms of different groups were generated by GraphPad Prism software Inc., San Diego, CA.

## Results

### MiRNA Identification and Expression Profile

All the clean reads were aligned to the small RNAs in the miRBase 22 database; the alignment rates for the 9 libraries with goat known miRNAs were all over 60%, and the alignment rates for other known miRNAs were all over 10%. All of the unannotated reads were aligned with the reference genome. According to their genome positions and hairpin structures predicted by Mireap_v0.2 software, novel miRNA candidates were identified. The alignment rates for novel miRNAs in the nine libraries were in the range of 0.04–0.1% (Supplementary Table 2). All data have been uploaded to the Gene Expression Omnibus (GEO) database under the accession number PRJNA702107.

According to the alignment results, miRNA annotation and identification were performed. In this study, 418 known miRNAs in goats, 1,292 known miRNAs in other species, and 479 new miRNAs were identified (Table 1). As shown in Table 2, among the known miRNAs in goats, the expression level of 26, 55, 90, and 247 miRNAs reached extremely high expression (TPM ≥ 5,000), high expression (5,000 > TPM ≥ 500), medium expression (500 > TPM ≥ 500), and low expression (50 > TPM ≥ 1), respectively. Among the known miRNAs in other species, the number of miRNAs that reached extremely high expression, high expression, medium expression, and low expression was 9, 31, 60, and 1,192, respectively. For the novel miRNAs, only two miRNAs reached medium expression, and the other 477 miRNAs had low expression. Among all the miRNAs, the 10 miRNA families with the highest expression levels were miR-26, miR-148, let-7, miR-30, miR-99, miR-200, miR-125, miR-143, miR-27, and miR-181. The proportions of the expression of these miRNAs in each group are shown in Supplementary Figure 1a, and their sequences are shown in Table 3. The five miRNAs with the highest average expression levels were all goat known miRNAs, as shown in Table 4, and they were chi-miR-26a-5p, chi-miR-148a-3p, chi-miR-143-3p, chi-miR-99a-5p, and chi-miR-27b-3p. Together, their expression accounted for nearly one-third of the expression of all miRNAs (33.21%).

**Table 1:** Reults of miRNA alignment.

**Sample**	**Goat known miRNAs**	**Other known miRNAs**	**Novel miRNAs**
A1-S	397	710	341
A2-S	385	715	359
A3-S	398	730	361
B3-S	380	561	238
B4-S	383	635	268
B5-S	384	696	268
C2-S	383	637	278
C3-S	371	564	251
C5-S	383	517	246
Sum	418	1,292	479

**Table 2:** Statistic of different expression types of miRNAs.

**Type**	**miRNAs reached extremely high expression (TPM ≥ 5,000)**	**miRNAs reached high expression(5,000 > TPM ≥ 500)**	**miRNAs reached medium expression (500 > TPM ≥ 50)**	**miRNAs reached low expression(50 > TPM ≥ 1)**
Number of goat known miRNAs	26	55	90	247
Number of other known miRNAs	9	31	60	1,192
Number of novel goat miRNAs	/	/	2	477

**Table 3:** The information of top 10 highest expressed miRNA families.

**ID**	**Length**	**Sequence**
miR-26	22	CCTGTTCTCCATTACTTGGCTC
miR-148	22	AAAGTTCTGAGACACTCCGACT
let-7	22	GGAGGTAGTTGTTTGTACAGTT
miR-30	22	CTTTCAGTCGGATGTTTACAGC
miR-99	23	CAAGCTCGCTTCTATGGGTCTGT
miR-200	23	AATACTGCCTGGTAATGATGACA
miR-125	22	ACAAGTCAGGCTCTTGGGACCT
miR-143	21	GGTGCAGTGCTGCATCTCTGG
miR-27	22	AGAGCTTAGCTGATTGGTGAAC
miR-181	22	ACCATCGACCGTTGATTGTACC

**Table 4:** The Expression level of the top 5 miRNAs with highest expression levels in different groups.

**ID**	**Length**	**A-TPM**	**B-TPM**	**C-TPM**
chi-miR-26a-5p	22	110,494.84	97,914.41	89,331.81
chi-miR-148a-3p	22	38,923.31	116,345.74	120,793.92
chi-miR-143-3p	22	40,345.34	43,319.13	33,841.23
chi-miR-99a-5p	21	33,641.97	38,501.86	35,423.66
chi-miR-27b-3p	21	25,397.65	40,586.29	38,428.30

The results of miRNA length statistical analysis showed that the miRNAs in each of the three developmental stages ranged in length from 20 to 24 nt, of which the 22 nt miRNAs accounted for more than 40, and 47.08% of these miRNAs were expressed in the SMGs of 24-month-old adult goats (Supplementary Figure 1c). The PCA results showed that the first principal component (44.3%) could clearly distinguish the difference between group A and the other two groups (Supplementary Figure 1b), the results may indicate the similar expression of miRNAs between group B and group C.

### Identification of Differentially Expressed miRNAs

**Figure 1:**
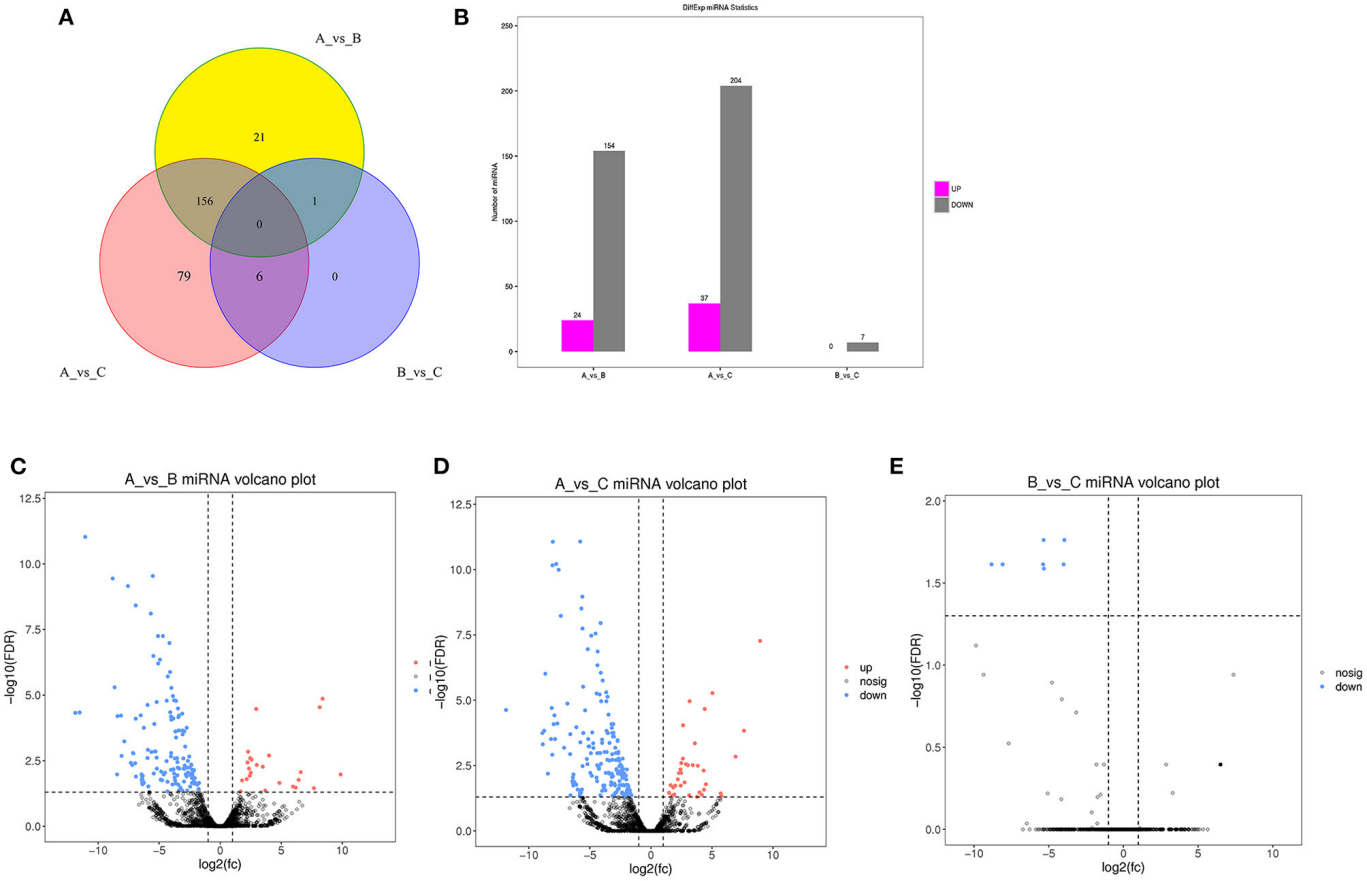
Identification of DE miRNAs. **(A)** Venn diagram of DE miRNAs between three different age groups. A were the samples for goats of 1-month-old (group A), B were the samples for goats of 12-month-old (group B), C were the samples for goats of 24-month-old (group C). **(B)** histogram of miRNAs at three different age groups. **(C)** Volcano Plot of DE miRNAs between group A and B. **(D)** Volcano plot of DE miRNAs between group A and C. **(E)** Volcano plot of DE miRNAs between group B and C.

The DE miRNA analysis revealed 178 and 241 DE miRNAs between group A and group B and between group A and group C, respectively, but only 7 DE miRNAs between group B and group C. These results further indicate the similar expression of miRNAs between group B and group C. It is worth noting that more than 80% of the DE miRNAs were significantly highly expressed in group A and expressed at low levels in groups B and C, and the results are shown in Figure 1.

Conservation analysis and species source analysis were performed on DE miRNAs. As shown in Supplementary Figure 2a, the top 20 miRNA families of all DE miRNAs with high expression were let-7, miR-30, miR-9, miR-181, miR-92, miR-29, miR-124, miR-125, miR-10, miR-7, miR-2, miR-133, miR-34, miR-19, miR-1, miR-26, miR-2285, miR-199, miR-135, and miR-219. Conservation analysisof the DE miRNAs showed that most of the DE miRNAs matched the species of *Homo sapiens, Bos taurus, Mus musculus*, with the identified miRNA number of 417, 391, and 358, respectively (Supplementary Figure 2d). To goat known DE miRNAs, the top 20 miRNA families with high expression were different from the other species known DE miRNA families. There were no miR-2 and miR-2285, except for miR-23 and miR-27 (Supplementary Figures 2b,c), and no *Gallus gallus* in the top 20 species sources, including *Pan paniscus* (Supplementary Figures 2e,f).

### Expression Pattern Analysis of DE miRNAs

**Figure 2:**
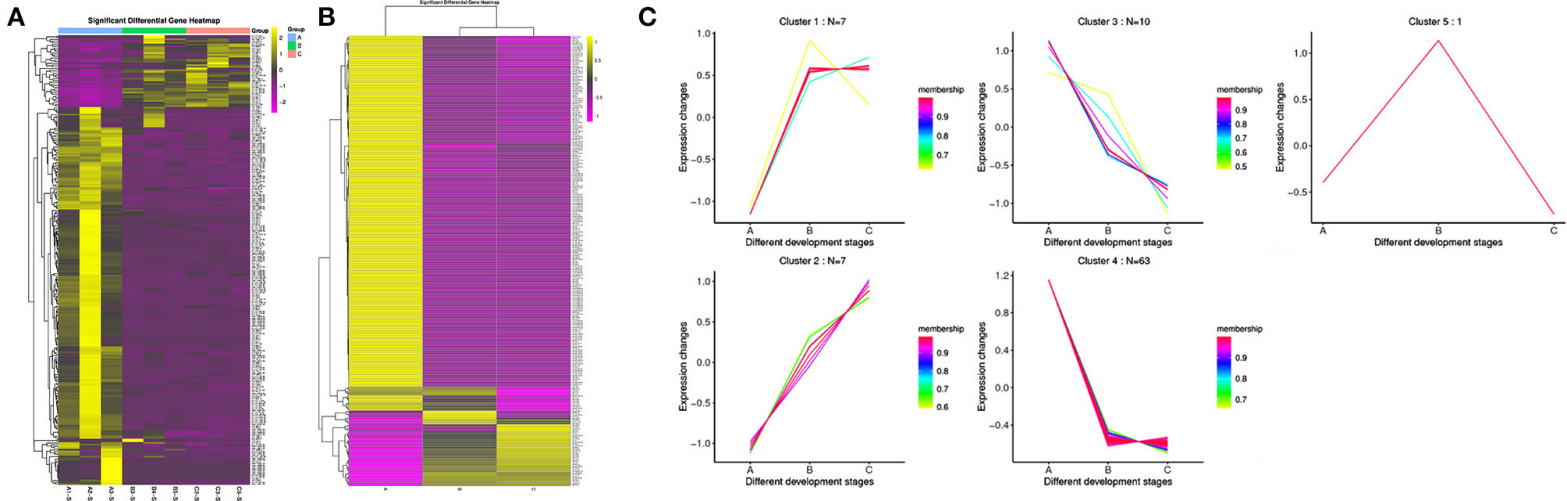
Clustering pattern of DE miRNAs. **(A)** Clustering pattern of DE miRNAs between different samples. A1, A2, A3 were the samples for 1-month-old kids (group A), B3, B4, B5 were the samples for 12-month-old maiden goats (group B), C2, C3, C5 were the samples for 24-month-old adult goats (group C). **(B)** Clustering pattern of DE miRNAs between different groups. A were the samples group A, B were the samples for group B, C were the samples for group C. **(C)** Trends of medium and above expression of DE miRNAs. Cluster 1 shows the DE miRNAs that lowly expressed in group A and highly expressed in group B and C. Cluster 2 shows the DE miRNAs that gradually increased in group A, B and C. Cluster 3 shows the DE miRNAs that gradually decreased in group A, B, and C. Cluster 4 shows the DE miRNAs that highly expressed in group A and lowly expressed in group B and C. Cluster 5 shows the DE miRNAs that highly expressed in group B and lowly expressed in group A and C.

The results showed that all the DE miRNAs were significantly clustered among the different groups (Figures 2A,B). Among all the DE miRNAs, we focused on the miRNAs whose expression levels reached a medium and high level. 59 and 86 DE miRNAs were identified between group A and B and group A and C, respectively. Only chi-miR-206 met the requirement between group B and C, and this result may be related to the small changes that occur in goat SMGs after puberty. We identified a total of 88 DE miRNAs after consolidation, including 54 known miRNAs of goats, 26 known DE miRNAs of other species and eight novel miRNAs. Expression pattern analysis was performed to confirm the changes in the expression patterns of these miRNAs in the three different age groups (Figure 2C). The results showed that the 88 DE miRNAs were grouped into 5 categories: cluster 1, cluster 2, cluster 3, cluster 4, and cluster 5. 7 miRNAs were included in cluster 1, and these miRNAs were expressed at low levels in group A and expressed at high levels in groups B and C. Cluster 2 also contained 7 miRNAs, and their expression gradually increased with growth and development. Ten miRNAs were included in cluster 3, and the expression trend was exactly opposite that of cluster 2; that is, the expression of these miRNAs gradually decreased with growth and development. Cluster 4 contained the largest number of miRNAs, 63, and these miRNAs were highly expressed in group A but expressed at low levels in groups B and C. Only one miRNA was included in cluster 5, that is, chi-miR-206, which was highly expressed in group B but expressed at low levels in groups A and C. The expression pattern results indicate that the expression of the DE miRNAs was time specific, and miRNAs with similar expression patterns may have similar functions.

### Target Gene Prediction and Functional Enrichment Analysis

To analyze the functions of miRNAs with medium and high expression levels and with different expression patterns, the target genes of the DE miRNAs in goat SMGs were predicted with the standard of −1.0 < Pearson correlation coefficient < -0.68 and *p*-value < 0.05. Then, GO and KEGG pathway enrichment analyses of the miRNA target genes with different expression patterns were performed.

**Figure 3:**
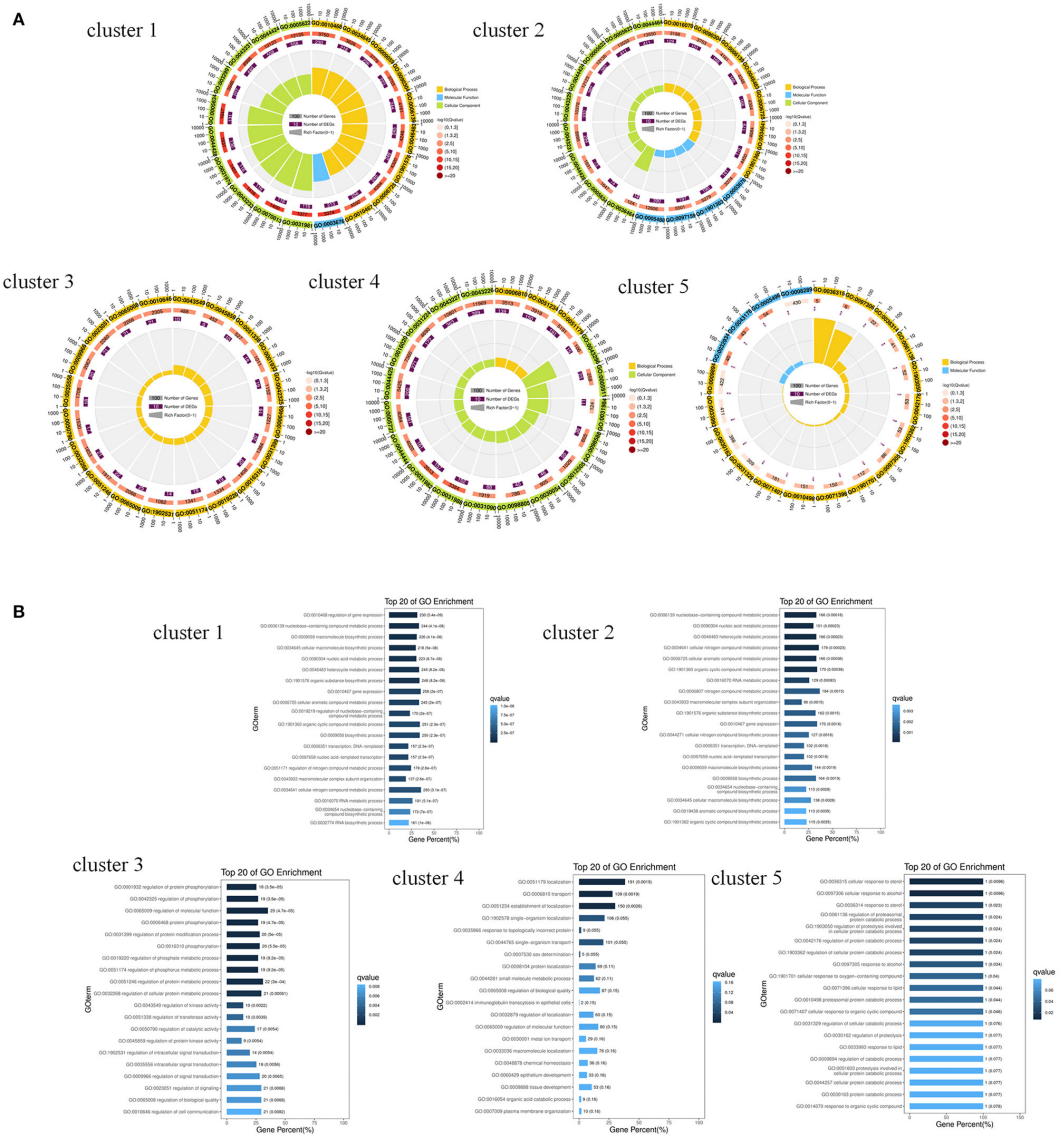
GO enrichment analysis of miRNA predicted target genes. **(A)** The GO enrichment analysis of target genes predicted by miRNAs with different expression patterns. **(B)** The biological process terms of GO enrichment analysis of target genes predicted by miRNAs with different expression patterns.

**Figure 4:**
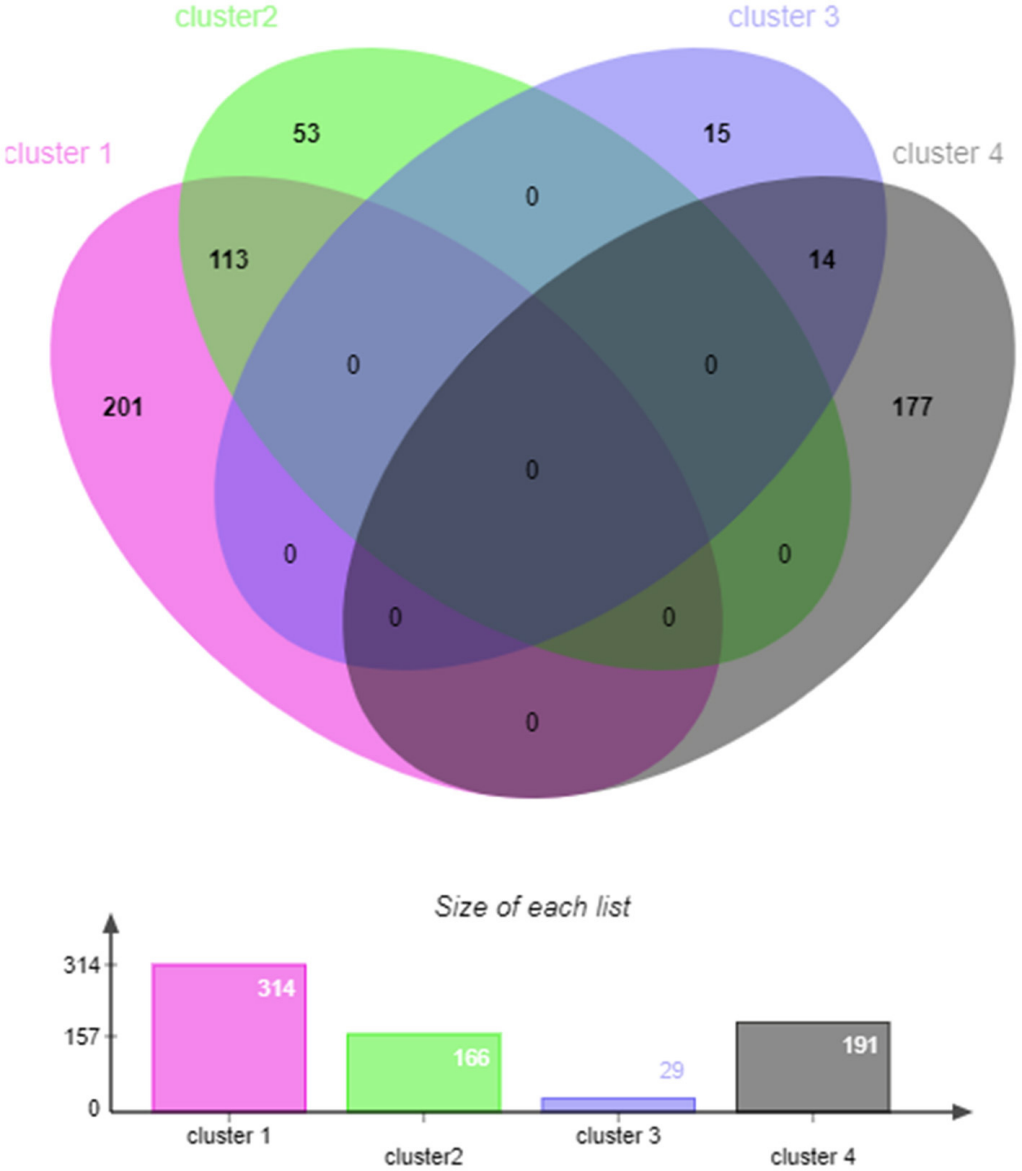
The venn diagram of target genes that significantly enriched in the first three GO terms in cluster 1–4.

GO enrichment analysis of the miRNA target genes with different expression patterns is shown in Figure 3. Among the significantly enriched terms of the five expression pattern clusters, more than half were enriched in biological processes. As shown in Figures 3B, 4 and Supplementary Table 3, most of the cluster 1–4 miRNA target genes that significantly enriched in the first three terms were different. These results suggested that the expression of differentially expressed genes in the SMGs of goats at 1, 12, and 24 months of age was regulated by miRNAs. With the growth and development of goats, the gene functions of SMG mRNAs were also altered by miRNA regulation.

**Figure 5:**
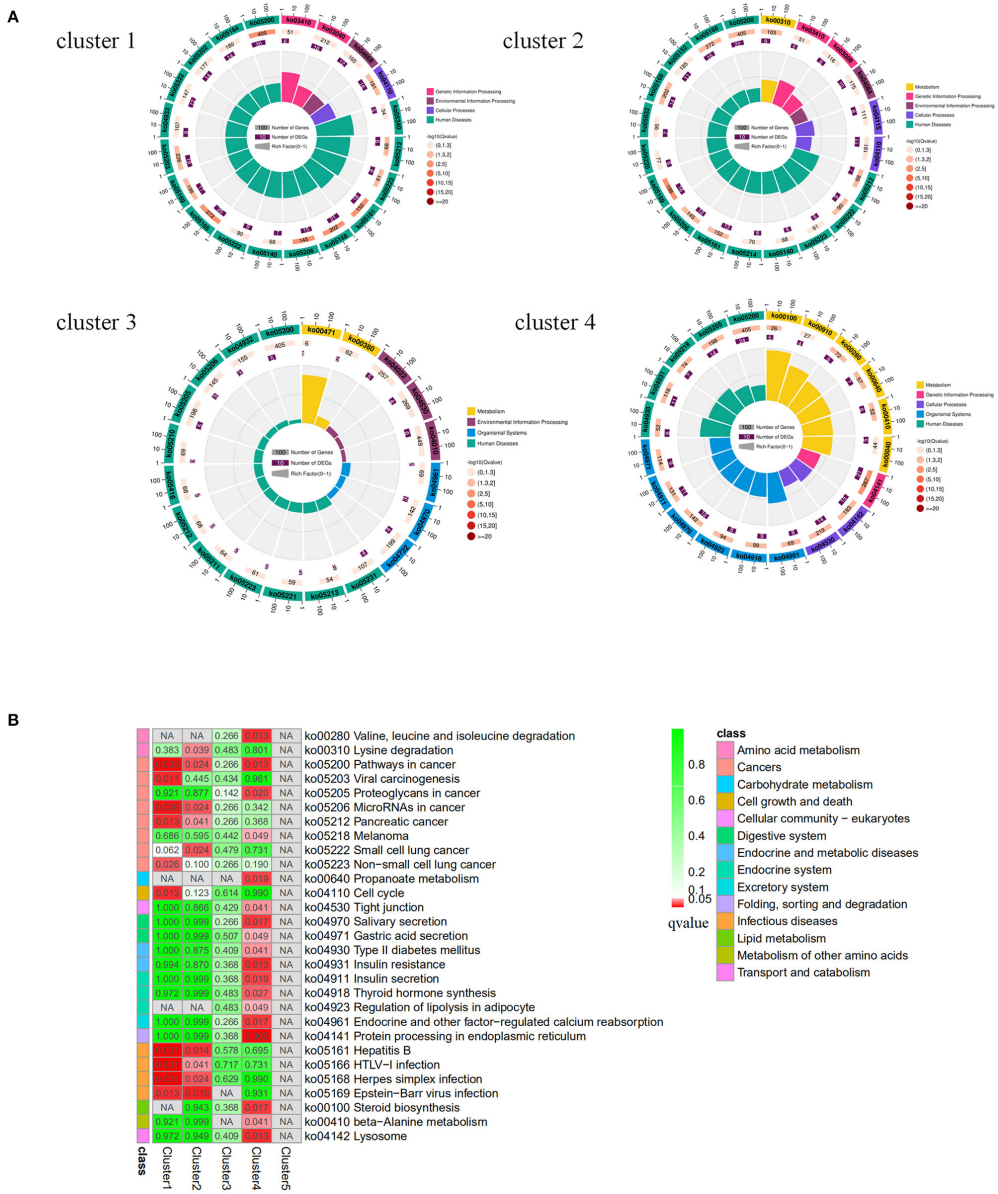
KEGG enrichment analysis of target genes predicted by different miRNA expression patterns. **(A)** Circular map of KEGG enrichment analysis results of target genes predicted by miRNAs with different expression patterns. **(B)** Comparison of target genes enriched pathways that were predicted by miRNAs with different expression patterns.

The results of the KEGG pathway enrichment analysis of target genes are shown in Figure 5. For Cluster 1 and 2, most of the significantly enriched pathways were related to disease, and the three most significantly enriched pathways in Cluster 1 were Hepatitis B (FDR = 0.00054), Herpes simplex infection (FDR = 0.00054), HTLV-I infection (FDR = 0.00054); the most significantly enriched pathways in cluster 2 were Epstein-Barr virus infection (FDR = 0.0024), Hepatitis B (FDR = 0.014), and MiRNAs in cancer (FDR = 0.024). The pathways that were not only significantly enriched in cluster 1 but also significantly enriched in cluster 2 were Epstein-Barr virus infection, Hepatitis B, Herpes simplex infection, HTLV-I infection, miRNAs in cancer, and Pancreatic cancer. For cluster 4, the three most significantly enriched pathways were Protein processing in the endoplasmic reticulum (FDR = 0.0024), Valine, leucine, and isoleucine degradation (FDR = 0.0133) and Lysosomes (FDR = 0.0133). This may indicate that the DE miRNAs expressed at low levels in the SMGs of 1-month-old goats were related to the occurrence of infectious diseases, while the DE miRNAs expressed at high levels were related to protein formation processes and amino acid metabolism or other pathways.

### Network Analysis

**Figure 6:**
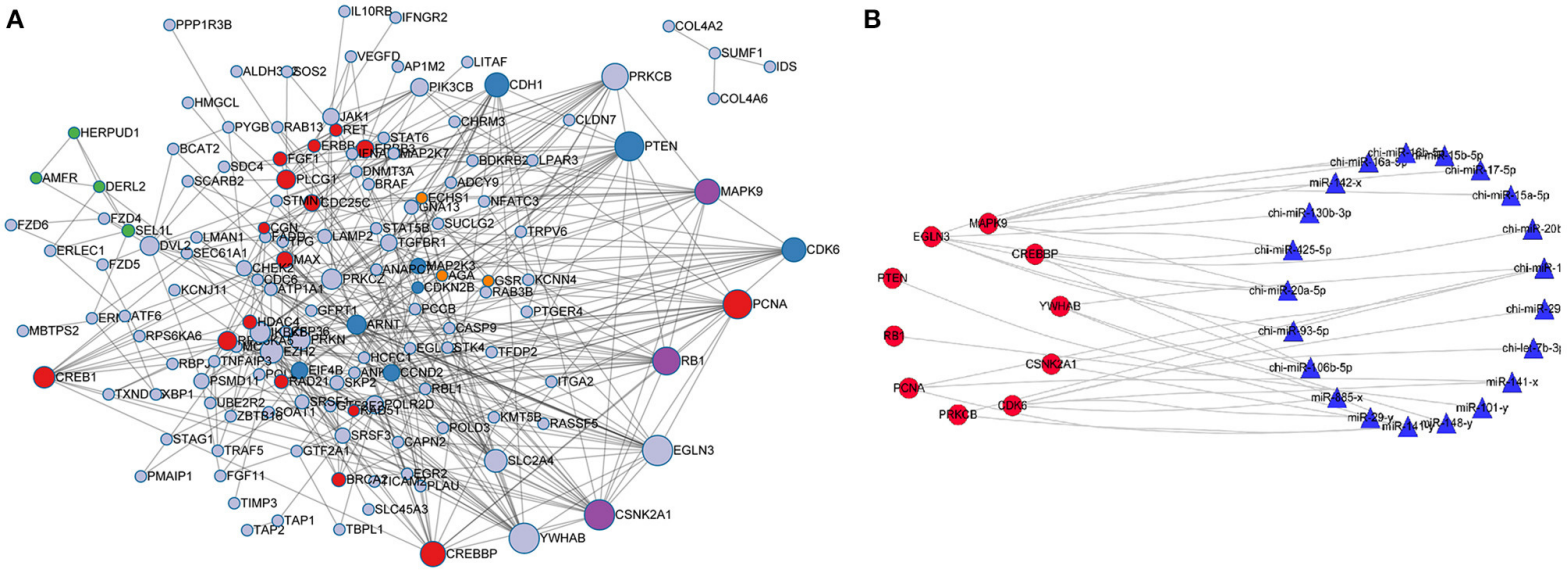
Protein-protein interaction network and network analysis for core genes and miRNAs. **(A)** PPI interaction network was constructed for the significantly enriched miRNA target genes. **(B)** Network analysis for core genes and miRNAs.

Protein-protein interaction analysis (PPI) was performed on 181 target genes that were significantly enriched in the KEGG pathways. The results are shown in Figure 6A. There were 612 interactions for 168 genes. All the genes in the network were ranked according to the degree of interaction, and the top 10 potential key target genes, namely, *CDK6, CSNK2A1, EGLN3, MAPK9, PCNA, PRKCB, PTEN, RB1, CREBBP*, and *YWHAB*, were predicted to be core genes. KEGG pathway enrichment analysis was further performed on the potential key target genes, and it was found that the significantly enriched pathways were Hepatitis B (FDR = 9.03E-10) and Pathways in cancer (FDR = 4.2E-10).

According to the screening criteria of Pearson correlation coefficient < -0.68 and *p*-value < 0.05, a total of 21 miRNAs were shown to target the 10 core target genes identified in the RNA-seq data of goat SMG miRNAs and mRNAs. Network analysis was performed with Cytoscape 3.7.2 with the 10 core target genes and 21 miRNAs, and the results are shown in Figure 6B. The miRNAs with the most target genes were miR-141-y and chi-miR-141, both of which targeted four genes, namely, CSNK2A1, PCNA, PRKCB, and YWHAB, followed by chi-miR-20a-5p and miR-29-y, which targeted three and two genes, respectively. The top 10 miRNAs were chosen as core miRNAs according to their expression levels, and their fold change from large to small with FDR <0.05. The results are shown in Tables 5, 6. The 10 miRNAs were miR-29-y, miR-141-y, miR-142-x, chi-miR-93-5p, chi-miR-20b, chi-miR-20a-5p, chi-miR-17-5p, chi-miR-16b-5p, chi-miR-15b-5p, and chi-miR-130b-3p. Among the 10 miRNAs, miR-29-y and miR-141-y were gradually overexpressed in three developmental stages, while the other eight miRNAs were all highly expressed in 1-month-old goat SMGs and expressed at low levels in 12- and 24-month-old goat SMGs. This result may suggest that the miRNAs highly expressed in 1-month-old goat SMGs are related to diseases, but the specific regulatory mechanism requires further verification.

**Table 5:** Expression of core miRNAs in different groups.

**miRNA**	**A TPM**	**B TPM**	**C TPM**	**A-vs.-B log2(fc)**	**FDR**	**A-vs.-C log2(fc)**	**FDR**	**B-vs.-C log2(fc)**	**FDR**	**Expression difference between groups**
miR-142-x	47583.02	1029.68	861.31	−5.53	2.89E-10	−5.79	8.50E-12	−0.26		Highly expressed in group A and lowly expressed in groups B and C
chi-miR-15b-5p	2699.08	167.88	131.22	−4.01	5.31E-06	−4.36	1.36E-07	−0.36	1.00	
chi-miR-17-5p	450.06	43.40	40.27	−3.37	6.01E-05	−3.48	3.52E-05	−0.11	1.00	
chi-miR-20a-5p	686.20	80.39	83.98	−3.09	2.22E-04	−3.03	2.42E-04	0.06	1.00	
chi-miR-16b-5p	3648.18	477.97	433.80	−2.93	2.39E-04	−3.07	8.25E-05	−0.14	1.00	
chi-miR-93-5p	691.81	94.86	80.05	−2.87	9.11E-04	−3.11	2.45E-04	−0.24	1.00	
miR-29-y	190.56	929.43	1182.46	2.29	1.43E-03	2.63	9.09E-05	0.35	1	Lowly expressed in groups B and Cand highly expressed in group A
chi-miR-141	4638.52	21953.52	22818.10	2.24	3.67E-03	2.30	6.13E-03	0.06	1.00	
miR-141-y	286.62	1292.21	1599.55	2.17	1.58E-02	2.48	6.01E-03	0.31	1.00	
miR-141-x	31.00	169.48	167.04	2.45	2.53E-03	2.43	4.40E-03	−0.02	1.00	

**Table 6:** Sequences of core miRNAs and their target genes.

**miRNA**	**Length**	**Sequence**	**mRNA**	**Symbol**
miR-142-x	21	CATAAAGTAGAAAGCACTACT	XM_005695224.3	EGLN3
chi-miR-15b-5p	22	TAGCAGCACATCATGGTTTACA	XM_018051618.1	MAPK9
chi-miR-17-5p	24	CAAAGTGCTTACAGTGCAGGTAGT	XM_018051618.1	MAPK9
chi-miR-20a-5p	23	TAAAGTGCTTATAGTGCAGGTAG	XM_005695224.3	EGLN3
			XM_018051618.1	MAPK9
			NM_001314227.1	SLC2A4
chi-miR-16b-5p	22	TAGCAGCACGTAAATATTGGGG	XM_018051618.1	MAPK9
chi-miR-93-5p	23	CAAAGTGCTGTTCGTGCAGGTAG	XM_005695224.3	EGLN3
miR-29-y	23	TAGCACCATCTGAAATCGGTTAA	XM_018047425.1	CDK6
			XM_005698175.3	PTEN
chi-miR-141	22	TAACACTGTCTGGTAAAGATGG	XM_018057913.1	YWHAB
miR-141-y	24	TAACACTGTCTGGTAAAGATGGCT	XM_018057913.1	YWHAB
			XM_018057661.1	CSNK2A1
			XM_013974872.2	PRKCB
			XM_005688167.3	PCNA
miR-141-x	22	CATCTTCCAGCACAGTGTTGGA	XM_018047425.1	CDK6
			XM_005688167.3	PCNA

### Experimental Validation

**Figure 7:**
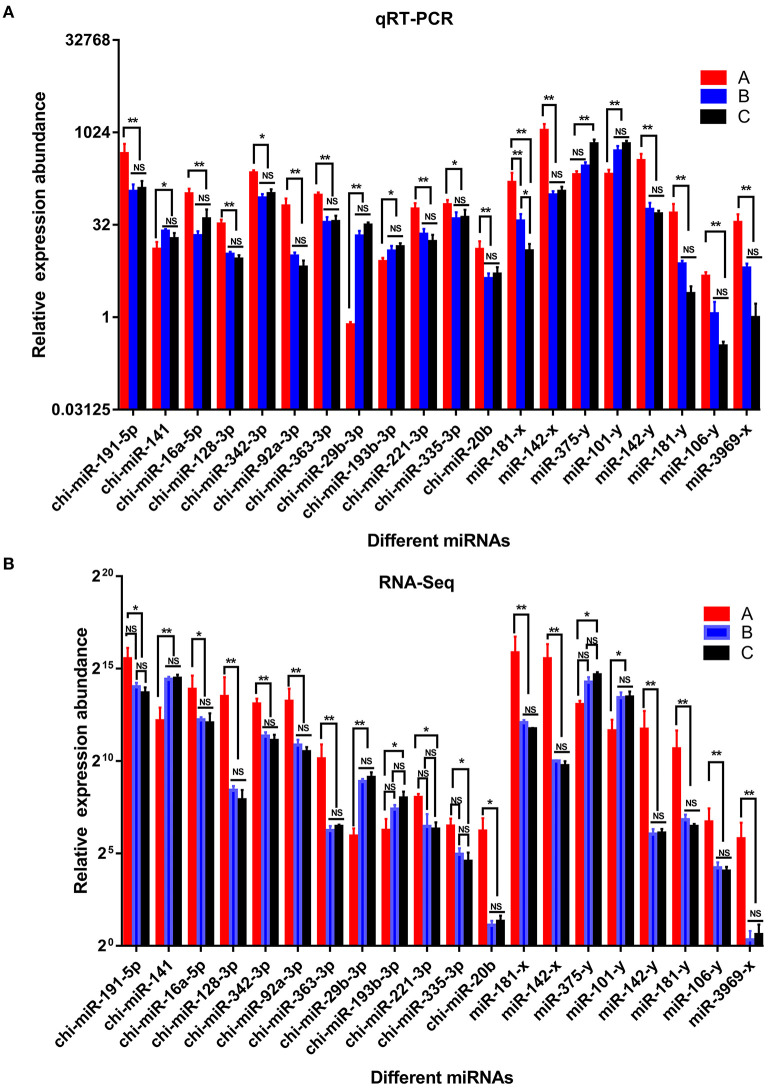
Comparison of relative expression of DE miRNAs between RNA-Seq and qRT-PCR results. **(A)** the results of qRT-PCR, **(B)** the results of RNA-seq. A were the samples for goat of 1-month-old kids (group A), B were the samples for goat of 12-month-old maiden goats goats (group B), C were the samples for goat of 24-month-old adult goats (group C). Significant differences are indicated by * and ** (* indicates *p* < 0.01, ** indicates *p* < 0.05), NS indicates there is no significant differences.

To verify the accuracy of the sequencing and analysis results, 20 DE miRNAs were selected for RT-qPCR validation (Figure 7). The qRT-PCR validation results for most of the selected miRNAs were consistent with the RNA-seq results.

## Discussion

In this study, we performed miRNA sequencing on the SMGs of goats at three different developmental stages. Currently, functional studies on salivary gland miRNAs are mainly focused on humans and mice. A number of studies have already investigated the role of multiple salivary gland miRNAs in the etiopathogenesis of Sjögren's syndrome (Argyropoulou et al., 2018; Reale et al., 2018). Mature miRNAs in the fetal salivary gland have also been found to affect the development of other tissues and organs by being loaded into exosomes. The above studies indicate that miRNAs play important roles in salivary gland development and functional maintenance. However, currently, reports on miRNA function in the salivary glands of ruminants are rare. To the best of our knowledge, this is the first miRNA sequencing and expression profiling study on goat and ruminant salivary glands.

The overexpression or underexpression of miRNAs affects gene expression, thus affecting the related pathways that may cause lesions. miRNAs are considered biomarkers of disease due to their ability to interfere with physiological or pathological processes. Some diseases can be prevented and diagnosed by the detection of miRNAs. As vertebrate-specific miRNAs, miR-26 family members have been detected in many different kinds of tumors and play important roles in cardiovascular disease, Parkinson's disease, neurodegenerative diseases, and many other non-tumor diseases (Gao and Liu, 2011; Icli et al., 2014; Goh et al., 2019; Su et al., 2019). The validated target genes of these miRNAs are involved in cell metabolism, proliferation, differentiation, apoptosis, autophagy, invasion, and metastasis (Lagos-Quintana et al., 2002; Li et al., 2020). In some tumors, the differential expression pattern of miR-26 might be related to specific tumorigenic processes. miR-125a-5p significantly inhibits the proliferation of head and neck cancer cells, whereas inhibition of miR-125a-5p enhances their proliferation (Xu et al., 2020). The expression of miR-181 family members is significantly related to colorectal cancer and correlated with the lymph node metastasis of oral squamous cell carcinoma, and miR-181 may play a role in the pathogenesis of Sjögren syndrome. The let-7 family was one of the first tumor suppressor miRNAs to be identified (Boyerinas et al., 2010). The upregulation of let-7 expression is associated with the progression and poor prognosis of hepatocellular carcinoma (Shi et al., 2017). let-7 as a regulator of myofibroblast differentiation through the regulation of IL-6 in mice (Di et al., 2021). Jiang et al. found that let-7 is expressed in a variety of immune cells, including B cells and macrophages, which indicates that let-7 may play physiological and pathological roles *in vivo* (Jiang, 2019). In human and murine glioblastoma/glioma, differentially expressed let-7 miRNAs are signaling molecules that induce microglial inflammatory cytokine release, modulate antigen presentation, and attenuate cell migration in a TLR7-dependent manner (Buonfiglioli et al., 2019; Song et al., 2020). During cardiac hypertrophy, the overexpression of miR-99 facilitates the transition from physiological hypertrophy to pathological hypertrophy, and silencing of miR-99 has the opposite effect, through the regulation of the Akt-1 pathway (Ramasamy et al., 2018). Renal clear cell carcinoma-derived miR-27a-loaded exosomes inhibit secreted frizzled-related protein 1 (SFRP1) expression and accelerate tumor angiogenesis (Hou et al., 2020). In Cr(VI)-induced carcinogenesis, miR-143 inhibits tumor growth and angiogenesis by regulating IL-6/HIF-1α *in vivo* (Wang et al., 2019). The miRNA-30 and miRNA-199 profiles in serum and tissue saliva samples distinguish benign from malignant salivary gland tumors (Cinpolat et al., 2017). In the SMGs of goats, most of these top 10 miRNA families are related to diseases, especially cancer. Among them, let-7, miR-30, miR-181, and miR-125 are differentially expressed RNAs, which may indicate their potential functions in SMGs with growth. This requires further study. Among the top 10 miRNA families, two are involved in some metabolic processes. miR-27a significantly affects the expression of mRNAs related to milk fat metabolism (Lin et al., 2013). miR-148a-3p inhibits alpaca melanocyte pigmentation by targeting MITF (Zhu et al., 2019).

In the present research, chi-miR-26a-5p, chi-miR-148a-3p, chi-miR-143-3p, chi-miR-99a-5p, and chi-miR-27b-3p were found to be the top five most highly expressed miRNAs. All five most highly expressed miRNAs were not differentially expressed among the three developmental stages, which may suggest that these miRNAs play important and stable roles in the SMGs of goat with growth. MiR-26a-5p was reported to be significantly downregulated in the B lymphocytes purified from primary Sjögren's syndrome patients (Wang-Renault et al., 2018). MiR-26a promoted endometrial epithelium cells proliferation and induced stromal cells apoptosis via the PTEN-PI3K/AKT pathway in dairy goats (Zhang et al., 2018). Dey et al. found that miR-26a is required for skeletal muscle differentiation and regeneration *in vivo* of mice. However, the function of miR-26a in the SMGs has not been reported (Dey et al., 2012). MiR-148a can negatively regulate cell proliferation, migration, and invasion by downregulating *MTA2* in salivary adenoid cystic carcinoma cells (Li et al., 2018). In goat rumen, the expression of miR-148a-3p was significantly different between stages, and involved in the development of the rumen epithelial cells by targeting *QKI*, meanwhile proliferation of *GES-1* cells were induced by miR148a-3p inhibitor (Zhong et al., 2020). So we hypothesize the stable expression of chi-miR-148a-3p in goat SMGs is also related to the growth and development of SMGs cells, which need further study. MiR-143-3p can affect epithelial cell homeostasis by targeting SERCA2b, RyR2, and AC9 in Sjogren's syndrome (Cortes et al., 2018). Chi-miR-143-3p influences the development of mammary gland epithelial cells in dairy goats via target *Ndfip1 in vitro* (Ji et al., 2019). MiR-99a-5p was found to play a role in the metastasis of salivary adenoid cystic carcinoma (Feng et al., 2017). Therefore, all five most highly expressed miRNAs except miR-27b-3p have already been reported to be involved in cell proliferation and apoptosis or homeostasis to maintain the salivary gland function. These miRNAs may play important roles in the salivary glands of goats and are not related to the differential regulation of transcriptional levels among different developmental stages. Their underexpression may cause pathological or physiological changes and lead to diseases.

DE miRNA analysis showed that there were more than 150 DE miRNAs between the SMGs of group A and group B and the SMGs of group A and group C; in addition, more than 80% of the DE miRNAs were significantly downregulated, while there were only seven DE miRNAs between the SMGs of group B and group C. The results indicated that miRNA expression in the SMGs significantly changed during the growth and development of goats from kids to adolescent goats, while miRNAs were steadily expressed in the SMGs from adolescent goats to adult goats. This suggested that miRNAs play a stable role in maintaining salivary gland function in goats after puberty.

To investigate the functions of the DE miRNAs with different expression profiles, expression pattern analysis was performed on the medium and highly expressed DE miRNAs, and five clusters were identified. The expression and profiles of the DE miRNAs were correlated with the developmental stages of the goats. GO and KEGG pathway enrichment analyses were performed on target genes in the five clusters. The results showed that the target genes of the miRNAs in the different clusters were significantly enriched in different GO terms and KEGG pathways. The target genes of the DE miRNAs, expressed at low levels in the SMGs of kids and highly expressed in the SMGs of adolescent and adult goats (clusters 1 and 2) were significantly enriched in infectious diseases, including Hepatitis B, Herpes simplex infection, and HTLV-I infection. The growth and development of the SMG is carried out under epithelial-mesenchymal interactions (Wang and Laurie, 2004), which are regulated by a complex of signaling molecules, including growth or differentiation factors, cell receptors and extracellular matrix (ECM) proteins. Activation of hepatic stellate cells, exaggerated expression of profibrogenic genes, and/or suppression of antifibrogenic genes can cause excessive and persistent accumulation of the ECM, which further leads to fibrosis (Wynn, 2008; Loureiro et al., 2020). This indicates that the target genes of the DE miRNAs in clusters 1 and 2 may be related to the growth and development of SMG ECM proteins and that their abnormal expression can cause infectious diseases. Epithelial-to-mesenchymal transition (EMT) is implicated in a wide array of malignant behaviors in cancer, including proliferation, invasion, and metastasis (Pan et al., 2021). Many studies have reported that miRNAs play a crucial role in the malignant behaviors of cancer cells (Volinia et al., 2010; Gianpiero and Carlo, 2013), including EMT-related cancer metastasis (Aiello and Kang, 2019), and some miRNAs have been predicted to be potential tumor biomarkers. We noticed that cancer-related pathways were significantly enriched by the target genes of the cluster 1 and 2 DE miRNAs of goat SMGs. Therefore, the cancer-related DE miRNAs in the goat SMGs may have potential diagnostic, prognostic, and therapeutic value in cancers, and this requires a better understanding. The target genes of the DE miRNAs that are highly expressed in the SMGs of kids and expressed at low levels in the SMGs of unbred adolescent and adult goats (cluster 4) were significantly enriched in metabolism-related and secretion-related pathways. Therefore, we speculated that the function of the goat SMGs changes after puberty compared with that at kids, and the SMGs mainly function in metabolism and secretion at later stages, which might be related to the gradual improvement of the goat immune system with growth and development (Jaskoll et al., 2001; Ghezzi and Ship, 2003). The results were basically consistent with our previous transcriptome study of goat SMGs, in which the genes that were highly expressed in the kid SMGs were significantly enriched in immune-related pathways (Wang et al., 2020). Therefore, we predicted that the biological function of goat SMGs changes with growth and development. The mechanism by which RNAs function in the goat SMGs is complex, and genes play their roles through the combined action of multiple types of RNAs.

Ten core miRNAs were screened by PPI analysis. The 10 core target genes were significantly enriched in Pathways of Hepatitis B (FDR = 9.03E-10) and Pathways in cancer (FDR = 4.2E-10), suggesting that miRNAs in the SMGs may be associated with the occurrence and progression of cancers and infectious diseases. Target gene prediction was performed on the 10 predicted core miRNAs, and the results showed that the expression of the target genes *CSNK2A1, PCNA, PrKCB, YWHAB*, and *CDK6* could be regulated by miRNAs expressed at low levels in the 1-month-old kids, namely, chi-miR-141, miR-141-x, miR-141-y, and miR-29-y. As a known target for tumor, casin kinase 2α1 (encoded by *CSNK2A1*) play key roles in cell cycle regulation, cellular differentiation, proliferation and apoptosis regulation (Tsuyuguchi et al., 2019). It's activation is required for transforming growth factor β-induced epithelial–mesenchymal transition (Kim et al., 2018). Proliferating cell nuclear antigen (PCNA) plays an essential role in controlling a variety of reactions as a component of the replication and repair machinery (Kelman, 1997; Maga and Hübscher, 2003). As a part of a transcription complex, CDK6 induces the expression of tumor suppressors (Kollmann et al., 2013; Sherr et al., 2016). Protein kinase Cβ (PRKCB) plays a role in autophagy regulation (Patergnani et al., 2013), CREBBP inactivation promotes the development of HDAC3-dependent lymphomas (Jiang et al., 2017), and abnormal expression of Casein kinase 2α1 (*CSNK2A1*) is linked to neurological diseases and cancer (Trinh et al., 2017). We predicted that chi-miR-141, miR-141-x, miR-141-y and miR-29-y mainly play a role in regulating the proliferation and differentiation of SMG cells. Our results also indicate that the growth and development of goat SMGs are basically completed before puberty. The expression of the target genes *MAPK9, CREBBP* and *EGLN3* could be regulated by the miRNAs chi-miR-15b-5p, chi-miR-16b-5p, chi-miR-17-5p, chi-miR-20a-5p, chi-miR-93-5p, and miR-142-x, which were highly expressed in the SMGs of 1-month-old kids and expressed at low levels in the SMGs of 12-month-old unbred adolesent goats and 24-month-old adult goats. JNK2 (encoded by *MAPK9*) is implicated in apoptosis (Sabapathy and Wagner, 2004; Dhanasekaran and Reddy, 2008; Wang et al., 2010). *EGLN3* and *CREBBP* also play important roles in the occurrence and development of disease, and *EGLN3* can promote cell apoptosis (Lee et al., 2005). The loss of *CREBBP* promotes the development of HDAC3-dependent lymphoma (Jiang et al., 2017). We predicted that chi-miR-15b-5p, chi-miR-16b-5p, chi-miR-17-5p, chi-miR-20a-5p, chi-miR-93-5p, and miR-142-x play a role in cell apoptosis of goat SMGs. Our results indicate that the fuction of goat SMGs was basically stable after puberty. Apoptosis has been considered as one of the main factors that may be related to loss of salivary gland secretory function (Hayashi, 2011). Apoptosis and inflammation are inseparably linked (Cullen et al., 2013).

In conclusion, a set of DE miRNAs in the SMGs of goats at different developmental stages were identified, and functional enrichment analysis showed that the target gene of them are mainly focused on cell proliferation, growth and apoptosis, and significantly related to disease. The DE miRNAs identified in our research might contribute to disease prevention and be involved in the gene regulation of submandibular glands at different developmental stages. Our results also indicated that the growth and development of goat SMGs are basically completed before puberty.

## Data Availability Statement

The datasets presented in this study can be found in online repositories. The names of the repository/repositories and accession number(s) can be found below: NCBI [Accession: PRJNA702107].

## Ethics Statement

The animal study was reviewed and approved by Committee of the Management and Use of Laboratory Animals of Shandong Agricultural University (Permit Number: 2,007,005).

## Author Contributions

JW, TC, and AW contributed to conception and design of the study. XZ and LH organized the database. RX performed the statistical analysis. AW wrote the first draft of the manuscript. QL and YC wrote sections of the manuscript. All authors contributed to manuscript revision, read, and approved the submitted version.

## Conflict of Interest

The authors declare that the research was conducted in the absence of any commercial or financial relationships that could be construed as a potential conflict of interest.

## Supplementary Material

Supplementary Figure 1 Top 10 unique miRNAs with highly expression levels, PCA analysis, and length distribution analyses of miRNAs. **(a)** The top 10 unique miRNAs with highly expression levels in goat submandibular glands. The unique miRNAs vs. their total copy number % of all unique miRNAs of each library. **(b)** The PCA analysis results of miRNAs. **(c)** Length distribution analysis of all the miRNAs.

Supplementary Figure 2 Analysis of DE miRNA family and species. **(a–c)** The number statistics of the top 20 miRNA families of all DE miRNAs, goat known DE miRNAs, and other spieces known miRNAs, respectively. **(d–f)** The number statistics of the top 20 source species of all DE miRNAs, goat known DE miRNAs, and other spieces known miRNAs, respectively.

Supplementary Table 1 The qRT-PCR primers used for validation.

Supplementary Table 2 Results of miRNA alignment.

Supplementary Table 3 Top three terms of Biological Process enrichment analysis of miRNA target genes with different expression patterns.

## References

[B1] Aiello N. M. Kang Y. (2019). Context-dependent EMT programs in cancer metastasis. J. Exp. Med. 216, 1016–1026. 10.1084/jem.20181827 30975895 PMC6504222

[B2] Alexa A. Rahnenführer J. (2009). Gene set enrichment analysis with topGO. Bioconduct. Improv. 27, 1–26. 24884810

[B3] Argyropoulou O. D. Valentini E. Ferro F. Leone M. C. Cafaro G. Bartoloni E.. (2018). One year in review 2018: Sjögren's syndrome. Clin. Exp. Rheumatol. 36, S14–S26. 30156536

[B4] Boyerinas B. Park S. M. Hau A. Murmann A. E. Peter M. E. (2010). The role of let-7 in cell differentiation and cancer. Endocr. Relat. Cancer. 17, F19–36. 10.1677/ERC-09-0184 19779035

[B5] Buonfiglioli A. Efe I. E. Guneykaya D. Ivanov A. Huang Y. Orlowski E.. (2019). let-7 microRNAs regulate microglial function and suppress glioma growth through Toll-like receptor 7. Cell Rep. 29, 3460–3471. 10.1016/j.celrep.2019.11.029 31825829

[B6] Chen L. Zhang T. Zhang S. Huang J. Zhang G. Xie K.. (2019). Identification of long non-coding RNA-associated competing endogenous RNA network in the differentiation of chicken preadipocytes. Genes 10:795. 10.3390/genes10100795 31614854 PMC6826404

[B7] Chen Z. Luo J. Ma L. Wang H. Cao W. Xu H. (2015). MiR130b-Regulation of PPARγ coactivator-1α suppresses fat metabolism in goat mammary epithelial cells. PLoS ONE. 10:e0142809. 10.1371/journal.pone.0142809 26579707 PMC4651502

[B8] Chen Z. Luo J. Sun S. Cao D. Shi H. Loor J. J. (2017). miR-148a and miR-17-5p synergistically regulate milk TAG synthesis via PPARGC1A and PPARA in goat mammary epithelial cells. RNA Biol. 14, 326–338. 10.1080/15476286.2016.1276149 28095188 PMC5367336

[B9] Chen Z. Shi H. Sun S. Xu H. Cao D. Luo J. (2016). MicroRNA-181b suppresses TAG via target IRS2 and regulating multiple genes in the Hippo pathway. Exp. Cell. Res. 348, 66–74. 10.1016/j.yexcr.2016.09.004 27616141

[B10] Chen Z. Xu X. Tan T. Chen D. Liang H. Sun K.. (2019). MicroRNA-145 regulates immune cytokines via targeting FSCN1 in *Staphylococcus aureus*-induced mastitis in dairy cows. Reprod. Domest. Anim. 54, 882–891. 10.1111/rda.13438 30974481

[B11] Cinpolat O. Unal Z. N. Ismi O. Gorur A. Unal M. (2017). Comparison of microRNA profiles between benign and malignant salivary gland tumors in tissue, blood and saliva samples: a prospective, case-control study. Braz. J. Otorhinolaryngol. 83, 276–284. 10.1016/j.bjorl.2016.03.013 27184509 PMC9444796

[B12] Cock P. J. Fields C. J. Goto N. Heuer M. L. Rice P. M. (2010). The Sanger FASTQ file format for sequences with quality scores, and the Solexa/Illumina FASTQ variants. Nucleic Acids Res. 38, 1767–1771. 10.1093/nar/gkp1137 20015970 PMC2847217

[B13] Cortes J. Tandon M. Jang S. Teos L. Alevizos I. (2018). MiR-143-3p targets calcium-transporting ATPase sarcoplasmic reticulum isoform 2b (SERCA2b), ryanodine receptor 2 (RyR2) and adenylylcyclase 9 (AC9) contributing to the loss of epithelial cell homeostasis in Sjogren's syndrome. Clin. Exp. Rheumatol. 36, S244–S245.

[B14] Cullen S. P. Henry C. M. Kearney C. J. Logue S. E. Feoktistova M. Tynan G. A.. (2013). Fas/CD95-induced chemokines can serve as “find-me” signals for apoptotic cells. Mol. Cell. 49, 1034–1048. 10.1016/j.molcel.2013.01.025 23434371

[B15] D'Avola T. E. Ogawa K. Alves e Silva M. R. Motoyama A. A. Inácio E. König Junior B.. (2006). Three-dimensional characteristics of submandibular salivary gland of ageing rats: an HRSEM study. Ann. Anat. 188, 431–438. 10.1016/j.aanat.2006.05.009 16999206

[B16] Dey B. K. Gagan J. Yan Z. Dutta A. (2012). miR-26a is required for skeletal muscle differentiation and regeneration in mice. Genes Dev. 26, 2180–2191. 10.1101/gad.198085.112 23028144 PMC3465739

[B17] Dhanasekaran D. N. Reddy E. P. (2008). JNK signaling in apoptosis. Oncogene 27, 6245–6251. 10.1038/onc.2008.301 18931691 PMC3063296

[B18] Di T. Yang Y. Fu C. Zhang Z. Qin C. Sai X.. (2021). Let-7 mediated airway remodelling in chronic obstructive pulmonary disease via the regulation of IL-6. Eur. J. Clin. Invest. 51:e13425. 10.1111/eci.13425 33037614 PMC7988621

[B19] Ekström J. Ekman R. Håkanson R. Luts A. Sundler F. (1994). Developmental studies on vasoactive intestinal peptide, substance P and calcitonin gene-related peptide in salivary glands of postnatal rats. Acta Physiol. Scand. 151, 107–115. 10.1111/j.1748-1716.1994.tb09726.x 7519388

[B20] Enright A John B. Gaul U. Tuschl T. Sander C. Marks D. (2003). MicroRNA targets in *Drosophila*. Genome Biol. 4, 1–27. 10.1186/gb-2003-5-1-r1 PMC395733 14709173

[B21] Feng X. Matsuo K. Zhang T. Hu Y. Mays A. C. Browne J. D.. (2017). MicroRNA profiling and target genes related to metastasis of salivary adenoid cystic carcinoma. Anticancer Res. 37, 3473–3481. 10.21873/anticanres.11715 28668836

[B22] Gao J. Liu Q. (2011). The role of miR-26 in tumors and normal tissues. Oncol. Lett. 2, 1019–1023. 10.3892/ol.2011.413 22848262 PMC3406571

[B23] Ghezzi E. M. Ship J. A. (2003). Aging and secretory reserve capacity of major salivary glands. J. Dent. Res. 82, 844–848. 10.1177/154405910308201016 14514768

[B24] Gianpiero D. L. Carlo M. C. (2013). miRNA profiling of cancer. Cur. Opin. Genet. Dev. 23, 3–11. 10.1016/j.gde.2013.01.004 PMC3632255 23465882

[B25] Goh S. Y. Chao Y. X. Dheen S. T. Tan E. K. Tay S. S. W. (2019). Role of microRNAs in parkinson's disease. Int. J. Mol. Sci. 20:5649. 10.3390/ijms20225649 PMC6888719 31718095

[B26] Hayashi T. (2011). Dysfunction of lacrimal and salivary glands in Sjogren's syndrome: nonimmunologic injury in preinflammatory phase and mouse model. J. Biomed. Biotechnol. 2011:407031. 10.1155/2011/407031 21660135 PMC3110304

[B27] Hiramatsu M. Kashimata M. Takayama F. Minami N. J. (1994). Developmental changes in and hormonal modulation of epidermal growth factor concentration in the rat submandibular gland. J. Endocrinol. 140, 357–363. 10.1677/joe.0.1400357 8182362

[B28] Hou Y. Fan L. Li H. (2020). Oncogenic miR-27a delivered by exosomes binds to SFRP1 and promotes angiogenesis in renal clear cell carcinoma. Mol. Ther. Nucleic Acids 24, 92–103. 10.1016/j.omtn.2020.11.019 33738141 PMC7941030

[B29] Hudder A. Novak R. F. (2008). miRNAs: effectors of environmental influences on gene expression and disease. Toxicol. Sci. 103, 228–240. 10.1093/toxsci/kfn033 18281715 PMC2922762

[B30] Icli B. Dorbala P. Feinberg M. W. (2014). An emerging role for the miR-26 family in cardiovascular disease. Trends Cardiovasc. Med. 24, 241–248. 10.1016/j.tcm.2014.06.003 25066487 PMC4150842

[B31] Jaskoll T. Chen H. Zhou Y. M. Wu D. Melnick M. (2001). Developmental expression of survivin during embryonic submandibular salivary gland development. BMC Dev. Biol. 1:5. 10.1186/1471-213X-1-5 11305929 PMC31339

[B32] Ji Z. He R. Chao T. Xuan R. Liu S. Wang G.. (2019). chi-miR-143-3p promotes apoptosis of mammary gland epithelial cells from dairy goats by targeting Ndfip1. DNA Cell Biol. 38, 1188–1196. 10.1089/dna.2019.4830 31603699

[B33] Ji Z. Liu Z. Chao T. Hou L. Fan R. He R.. (2017). Screening of miRNA profiles and construction of regulation networks in early and late lactation of dairy goat mammary glands. Sci. Rep. 7:11933. 10.1038/s41598-017-12297-4 28931951 PMC5607250

[B34] Ji Z. Wang G. Xie Z. Zhang C. Wang J. M. (2012). Identification and characterization of microRNA in the dairy goat (*Capra hircus*) mammary gland by Solexa deep-sequencing technology. Mol. Biol. Rep. 39, 9361–9371. 10.1007/s11033-012-1779-5 22763736

[B35] Jiang S. (2019). A regulator of metabolic reprogramming: microRNA let-7. Transl. Oncol. 12, 1005–1013. 10.1016/j.tranon.2019.04.013 31128429 PMC6531867

[B36] Jiang Y. Ortega-Molina A. Geng H. Ying H. Y. Hatzi K. Parsa S.. (2017). CREBBP inactivation promotes the development of HDAC3-dependent lymphomas. Cancer Discov. 7, 38–53. 10.1158/2159-8290.CD-16-0975 27733359 PMC5300005

[B37] Kameyama A. Thet Tin W. W. T. Nishijima R. Yamakoshi K. (2020). Alteration of mucins in the submandibular gland during aging in mice. Arch. Oral Biol. 121:104967. 10.1016/j.archoralbio.2020.104967 33197804

[B38] Kelman Z. (1997). PCNA: structure, functions and interactions. Oncogene. 14, 629–640. 10.1038/sj.onc.1200886 9038370

[B39] Kim S. Ham S. Yang K. Kim K. (2018). Protein kinase CK2 activation is required for transforming growth factor β-induced epithelial-mesenchymal transition. Mol. Oncol. 12, 1811–1826. 10.1002/1878-0261.12378 30171795 PMC6165993

[B40] Kollmann K. Heller G. Schneckenleithner C. Warsch W. Scheicher R. Ott R. G. (2013). A kinase-independent function of CDK6 links the cell cycle to tumor angiogenesis. Cancer Cell 24, 167–181. 10.1016/j.ccr.2013.07.012 23948297 PMC3743049

[B41] Krüger J. Rehmsmeier M. (2006). RNAhybrid: microRNA target prediction easy, fast and flexible. Nucleic Acids Res. 34, W451–W454. 10.1093/nar/gkl243 16845047 PMC1538877

[B42] Lagos-Quintana M. Rauhut R. Yalcin A. Meyer J. Lendeckel W. Tuschl T. (2002). Identification of tissue-specific microRNAs from mouse. Curr. Biol. 12, 735–739. 10.1016/S0960-9822(02)00809-6 12007417

[B43] Langmead B. Salzberg S. L. (2012). Fast gapped-read alignment with Bowtie 2. Nat. Methods 9:357. 10.1038/nmeth.1923 22388286 PMC3322381

[B44] Lee S. Nakamura E. Yang H. Wei W. Linggi M. S. Sajan M. P. (2005). NeuronalIdentification of tissue-specific microRNAs from mouse apoptosis linked to EglN3 prolyl hydroxylase and familial pheochromocytoma genes: developmental culling and cancer. Cancer Cell 8, 155–167. 10.1016/j.ccr.2005.06.015 16098468

[B45] Li C. Li Y Lu Y. Niu Z. Zhao H. Peng Y.. (2020). miR-26 family and its target genes in tumorigenesis and development. Crit. Rev. Oncol. Hematol. 157:103124. 10.1016/j.critrevonc.2020.103124 33254041

[B46] Li D. Zhan S. Wang Y. Wang L. Zhong T. Li L.. (2015). Role of microRNA-101a in the regulation of goat skeletal muscle satellite cell proliferation and differentiation. Gene 572, 198–204. 10.1016/j.gene.2015.07.010 26160440

[B47] Li L. Wang Y. Zhou X. (2018). Tumor-suppressive effect of miR-148a on salivary adenoid cystic carcinoma via down-regulation of MTA2. Int. J. Clin. Exp. Med. 11, 11973–11980.

[B48] Li M. Yu M. Liu C. Zhu H. He X. Peng S.. (2013). miR-34c works downstream of p53 leading to dairy goat male germline stem-cell (mGSC s) apoptosis. Cell Prolif. 46, 223–231. 10.1111/cpr.12013 23510477 PMC6495960

[B49] Liao R. Lv Y. Zhu L. Lin Y. (2019). Altered expression of miRNAs and mRNAs reveals the potential regulatory role of miRNAs in the developmental process of early weaned goats. PLoS ONE 14:e0220907. 10.1371/journal.pone.0220907 31393969 PMC6687162

[B50] Lin X. Luo J. Zhang L. Wang W. Shi H. Zhu J. (2013). MiR-27a suppresses triglyceride accumulation and affects gene mRNA expression associated with fat metabolism in dairy goat mammary gland epithelial cells. Gene 521, 15–23. 10.1016/j.gene.2013.03.050 23537996

[B51] Livak K. J. Schmittgen T. D. (2001). Analysis of relative gene expression data using real-time quantitative PCR and the 2– ΔΔCT method. Methods 25, 402–408. 10.1006/meth.2001.1262 11846609

[B52] Longpre K. M. Kinstlinger N. S. Mead E. A. Wang Y. Thekkumthala A. P. Carreno K. A.. (2014). Seasonal variation of urinary microRNA expression in male goats (Capra hircus) as assessed by next generation sequencing. Gen. Comp. Endocrinol. 199, 1–15. 10.1016/j.ygcen.2014.01.002 24457251

[B53] Loureiro D. Tout I. Narguet S. Benazzouz S. Mansouri A. Asselah T. (2020). miRNAs as potential biomarkers for viral hepatitis B and C. Viruses 12:1440. 10.3390/v12121440 33327640 PMC7765125

[B54] Love M. Anders S. Huber W. (2014). Differential analysis of count data–the DESeq2 package. Genome Biol. 15:550. 10.1186/s13059-014-0550-8 25516281 PMC4302049

[B55] Maga G. Hübscher U. (2003). Proliferating cell nuclear antigen (PCNA): a dancer with many partners. J. Cell. Sci. 116, 3051–3060. 10.1242/jcs.00653 12829735

[B56] Melnik B. C. Schmitz G. J. B. P. (2017). MicroRNAs: milk's epigenetic regulators. Best Pract. Res. Clin. Endocrinol. Metab. 31, 427–442. 10.1016/j.beem.2017.10.003 29221571

[B57] Na R. S. E G. X. Sun W. Sun X. W. Qiu X. Y. Chen L. P.. (2015). Expressional analysis of immune-related miRNAs in breast milk. Genet. Mol. Res. 14, 11371–11376. 10.4238/2015.September.25.4 26436378

[B58] Niu B. Wu J. Mu H Li B. Wu C. He X.. (2016). miR-204 regulates the proliferation of dairy goat spermatogonial stem cells via targeting to Sirt1. Rejuvenation Res. 19, 120–130. 10.1089/rej.2015.1719 26213858

[B59] Pan G. Liu Y. Shang L. Zhou F. Yang S. (2021). EMT-associated microRNAs and their roles in cancer stemness and drug resistance. Cancer Commun. 41, 199–217. 10.1002/cac2.12138 33506604 PMC7968884

[B60] Patergnani S. Marchi S. Rimessi A. Bonora M. Giorgi C. Mehta K. D. (2013). PRKCB/protein kinase C, beta and the mitochondrial axis as key regulators of autophagy. Autophagy 9, 1367–1385. 10.4161/auto.25239 23778835

[B61] Penschow J. D. Giles M. E. Coghlan J. P. Fernley R. T. (1997). Redistribution of carbonic anhydrase VI expression from ducts to acini during development of ovine parotid and submandibular glands. Histochem. Cell Biol. 107, 417–422. 10.1007/s004180050128 9208333

[B62] Ramasamy S. Velmurugan G. Rekha B. Anusha S. Rajan K. S. Shanmugarajan S.. (2018). Egr-1 mediated cardiac miR-99 family expression diverges physiological hypertrophy from pathological hypertrophy. Exp. Cell Res. 365, 46–56. 10.1016/j.yexcr.2018.02.016 29481791

[B63] Reale M. D'Angelo C. Costantini E. Laus M. Moretti A. Croce A. (2018). MicroRNA in Sjögren's syndrome: their potential roles in pathogenesis and diagnosis. J. Immunol. Res. 2018:7510174. 10.1155/2018/7510174 29977932 PMC6011049

[B64] Roy S. Sen C. K. (2011). MiRNA in innate immune responses: novel players in wound inflammation. Physiol. Genom. 43, 557–565. 10.1152/physiolgenomics.00160.2010 21139022 PMC3110889

[B65] Sabapathy K. Wagner E. F. (2004). JNK2: a negative regulator of cellular proliferation. Cell Cycle 3, 1520–1523. 10.4161/cc.3.12.1315 15611655

[B66] Schober P. Boer C. Schwarte L. A. (2018). Correlation coefficients: appropriate use and interpretation. Anesth. Analg. 126, 1763–1768. 10.1213/ANE.0000000000002864 29481436

[B67] Seeley J. J. Baker R. G. Mohamed G. Bruns T. Hayden M. S. Deshmukh S. D.. (2018). Induction of innate immune memory via microRNA targeting of chromatin remodelling factors. Nature 559, 114–119. 10.1038/s41586-018-0253-5 29950719 PMC6044474

[B68] Sethupathy P. Megraw M. Hatzigeorgiou A. G. (2006). A guide through present computational approaches for the identification of mammalian microRNA targets. Nat. Methods 3, 881–886. 10.1038/nmeth954 17060911

[B69] Sherr C. J. Beach D. Shapiro G. I. (2016). Targeting CDK4 and CDK6: from discovery to therapy. Cancer Discov. 6, 353–367. 10.1158/2159-8290.CD-15-0894 26658964 PMC4821753

[B70] Shi W. Zhang Z. Yang B. Guo H. Jing L. Liu T.. (2017). Overexpression of microRNA let-7 correlates with disease progression and poor prognosis in hepatocellular carcinoma. Medicine 96:e7764. 10.1097/MD.0000000000007764 28796071 PMC5556237

[B71] Song X. Liang Y. Sang Y. Li Y. Zhang H. Chen B.. (2020). CircHMCU promotes proliferation and metastasis of breast cancer by sponging let-7 family. Mol. Therapy Nucleic Acids 20, 518–533. 10.1016/j.omtn.2020.03.014 32330870 PMC7178009

[B72] Song Y. An X. Zhang L. Fu M. Peng J. Han P.. (2015). Identification and profiling of microRNAs in goat endometrium during embryo implantation. PLoS ONE 10:e0122202. 10.1371/journal.pone.0122202 25886011 PMC4401794

[B73] Su Y. Deng M. Xiong W. Xie A. Guo J. Liang Z.. (2019). MicroRNA-26a/death -associated protein kinase 1 signaling induces synucleinopathy and dopaminergic neuron degeneration in Parkinson's disease. Biol. Psychiatry 85, 769–781. 10.1016/j.biopsych.2018.12.008 30718039 PMC8861874

[B74] Taganov K. D. Boldin M. P. Chang K. J. Baltimore D. J. (2006). NF-κB-dependent induction of microRNA miR-146, an inhibitor targeted to signaling proteins of innate immune responses. PNAS 103, 12481–12486. 10.1073/pnas.0605298103 16885212 PMC1567904

[B75] Trinh J. Hüning I. Budler N. Hingst V. Lohmann K. Gillessen-Kaesbach G. (2017). A novel *de novo* mutation in CSNK2A1: reinforcing the link to neurodevelopmental abnormalities and dysmorphic features. J. Hum. Genet. 62, 1005–1006. 10.1038/jhg.2017.73 28725024

[B76] Tsuyuguchi M. Nakaniwa T. Sawa M. Nakanishi I. Kinoshita T. (2019). A promiscuous kinase inhibitor delineates the conspicuous structural features of protein kinase CK2a1. Acta Crystallograph. Sect. F 75, 515–519. 10.1107/S2053230X19008951 31282872 PMC6613443

[B77] Tucker A. (2007). Salivary gland development. Sem. Cell Dev. Biol. 18, 237–244. 10.1016/j.semcdb.2007.01.006 17336109

[B78] Turner M. Vigorito E. (2008). Regulation of B-and T-cell differentiation by a single microRNA. Biochem. Soc. Trans. 36, 531–533. 10.1042/BST0360531 18481999

[B79] Volinia S. Galasso M. Costinean S. Tagliavini L. Gamberoni G. Drusco A.. (2010). Reprogramming of miRNA networks in cancer and leukemia. Genome Res. 20, 589–599. 10.1101/gr.098046.109 20439436 PMC2860161

[B80] Wang A. Chao T. Ji Z. Xuan R. Liu S. Guo M.. (2020). Transcriptome analysis reveals potential immune function-related regulatory genes/pathways of female Lubo goat submandibular glands at different developmental stages. PeerJ. 8:e9947. 10.7717/peerj.9947 33083113 PMC7547598

[B81] Wang J. Laurie G. W. (2004). Organogenesis of the exocrine gland. Dev. Biol. 273, 1–22. 10.1016/j.ydbio.2004.05.025 15302594

[B82] Wang L. Qiu J. G. He J. Liu W. J. Ge X. Zhou F. M.. (2019). Suppression of miR-143 contributes to overexpression of IL-6, HIF-1α and NF-κB p65 in Cr (VI)-induced human exposure and tumor growth. Toxicol. Appl. Pharmacol. 378:114603. 10.1016/j.taap.2019.114603 31152816 PMC6897354

[B83] Wang P. Xiong Y. Ma C. Shi T. Ma D. (2010). Molecular cloning and characterization of novel human JNK2 (MAPK9) transcript variants that show different stimulation activities on AP-1. BMB Rep. 43, 738–743. 10.5483/BMBRep.2010.43.11.738 21110917

[B84] Wang-Renault S. F. Boudaoud S. Nocturne G. Roche E. Sigrist N. Daviau C.. (2018). Deregulation of microRNA expression in purified T and B lymphocytes from patients with primary Sjögren's syndrome. Ann. Rheum. Dis. 77, 133–140. 10.1136/annrheumdis-2017-211417 28916716 PMC5754740

[B85] Wu J. Zhu H. Song W Li M. Liu C. Li N.. (2014). Identification of conservative microRNAs in Saanen dairy goat testis through deep sequencing. Reprod. Domest. Anim. 49, 32–40. 10.1111/rda.12217 23981187

[B86] Wynn T. A. (2008). Cellular and molecular mechanisms of fibrosis. J. Pathol. 214, 199–210. 10.1002/path.2277 18161745 PMC2693329

[B87] Xiao C. Rajewsky K. (2009). MicroRNA control in the immune system: basic principles. Cell 136, 26–36. 10.1016/j.cell.2008.12.027 19135886

[B88] Xu M. Zhan J. Xie J. Zhu L. Chen L. Luo X.. (2020). MiR-125a-5p inhibits cell proliferation, cell cycle progression, and migration while promoting apoptosis in head and neck cancers by targeting ERBB3. Auris. Nasus. Larynx 48, 477–486. 10.1016/j.anl.2020.10.001 33077307

[B89] Xuan R. Chao T. Wang A. Zhang F. Sun P. Liu S.. (2020). Characterization of microRNA profiles in the mammary gland tissue of dairy goats at the late lactation, dry period and late gestation stages. PLos ONE 15:e0234427. 10.1371/journal.pone.0234427 32511270 PMC7279595

[B90] Yang N. Berry A. Sauer C. Baxter M. Donaldson I. J. Forbes K.. (2020). Hypoxia regulates GR function through multiple mechanisms involving microRNAs 103 and 107. Auris Nasus Larynx 518:111007. 10.1016/j.mce.2020.111007 32871225 PMC7646191

[B91] Yu G. Wang L. Han Y. He Q. (2012). clusterProfiler: an R package for comparing biological themes among gene clusters. Omics 16, 284–287. 10.1089/omi.2011.0118 22455463 PMC3339379

[B92] Yuan C. Wang X. Geng R. He X. Qu L. Chen Y. (2013). Discovery of cashmere goat (Capra hircus) microRNAs in skin and hair follicles by Solexa sequencing. BMC Genom. 14, 1–10. 10.1186/1471-2164-14-511 23889850 PMC3765263

[B93] Zhang L. Liu X. Liu J. Ma X. Zhou Z. Song Y.. (2018). miR-26a promoted endometrial epithelium cells (EECs) proliferation and induced stromal cells (ESCs) apoptosis via the PTEN-PI3K/AKT pathway in dairy goats. J. Cell. Physiol. 233, 4688–4706. 10.1002/jcp.26252 29115668

[B94] Zhong T. Wang C. Hu J. Chen X. Niu L. Zhan S.. (2020). Comparison of MicroRNA transcriptomes reveals the association between MiR-148a-3p expression and rumen development in goats. Animals 10:1951. 10.3390/ani10111951 33114089 PMC7690783

[B95] Zhu Z. Cai Y. Li Y. Li H. Zhang L. Xu D.. (2019). miR-148a-3p inhibits alpaca melanocyte pigmentation by targeting MITF. Small Rumin. Res. 177, 44–49. 10.1016/j.smallrumres.2019.06.004

