# Supplementary figures and images for: Differentially Expressed MiRNAs of Goat Submandibular Glands Among Three Developmental Stages Are Involved in Immune Functions
Source: Front Genet. 2021 Jun 15;12:678194. doi: 10.3389/fgene.2021.678194 (PMC8239366; doi:10.3389/fgene.2021.678194)

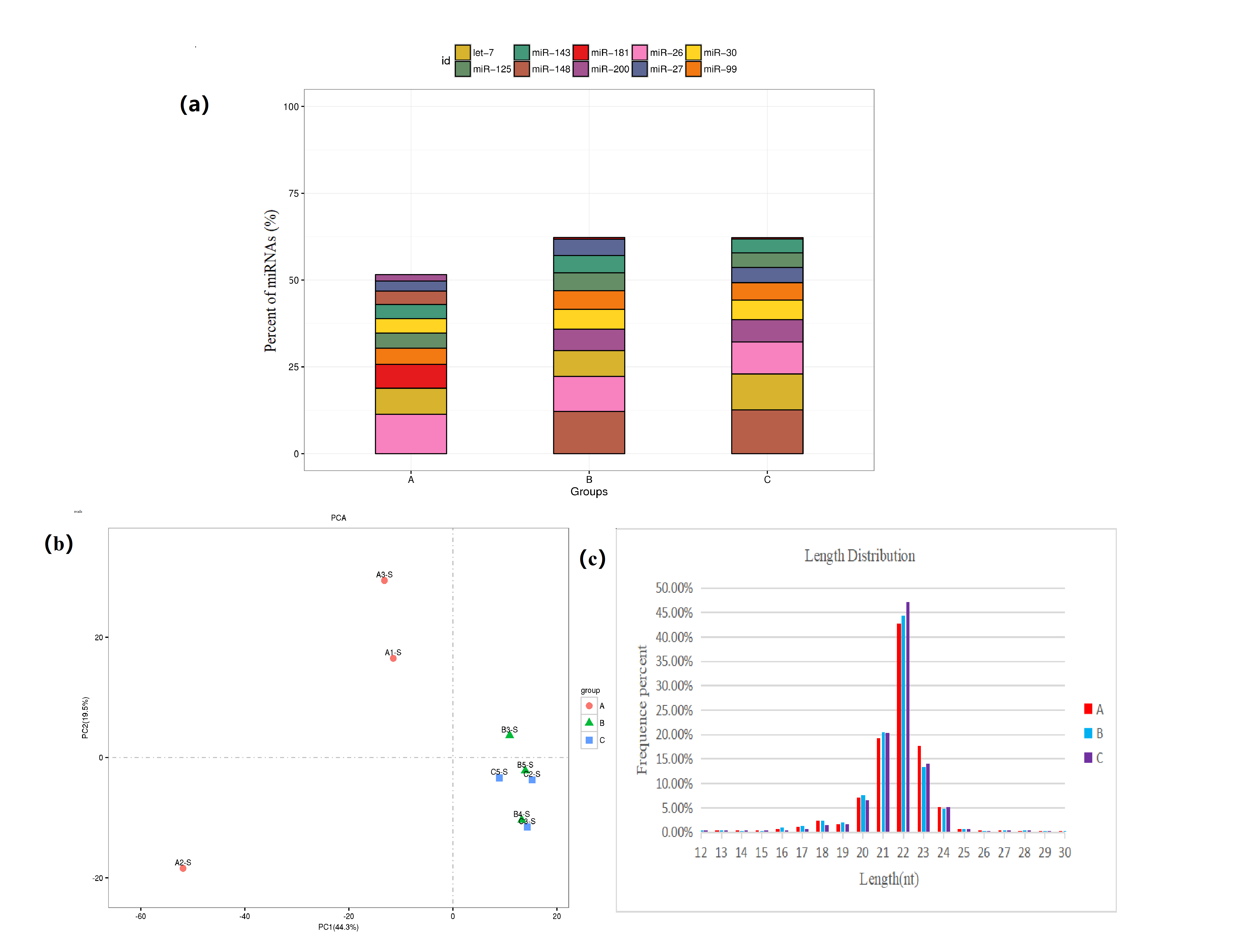

Supplement: Supplementary Figure 1 — Top 10 unique miRNAs with highly expression levels, PCA analysis, and length distribution analyses of miRNAs. (a) The top 10 unique miRNAs with highly expression levels in goat submandibular glands. The unique miRNAs vs. their total copy number % of all unique miRNAs of each library. (b) The PCA analysis results of miRNAs. (c) Length distribution analysis of all the miRNAs. [file Image_1.TIF]

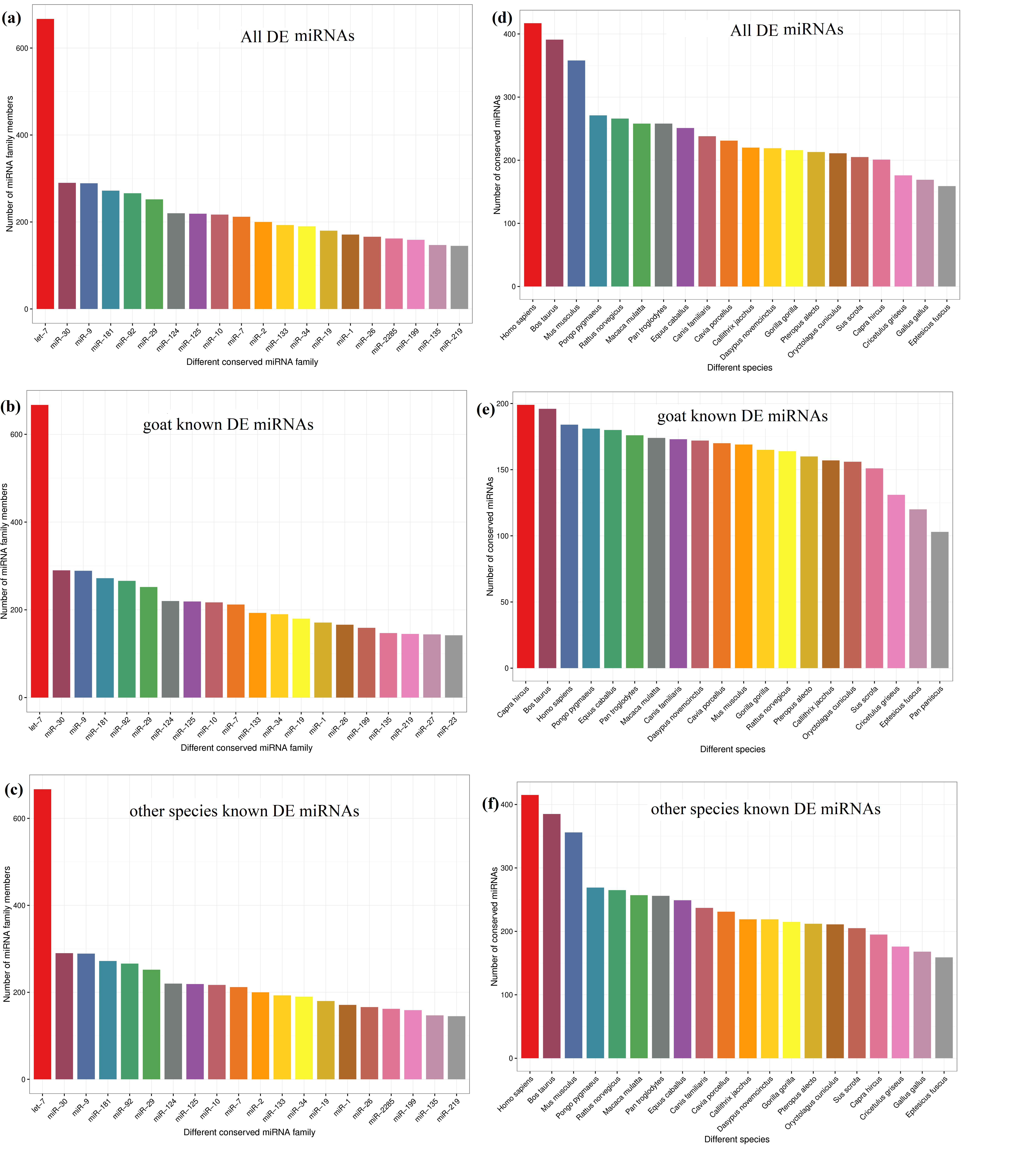

Supplement: Supplementary Figure 2 — Analysis of DE miRNA family and species. (a–c) The number statistics of the top 20 miRNA families of all DE miRNAs, goat known DE miRNAs, and other spieces known miRNAs, respectively. (d–f) The number statistics of the top 20 source species of all DE miRNAs, goat known DE miRNAs, and other spieces known miRNAs, respectively. [file Image_2.TIF]
