# Supplementary material for: Differentially Expressed MiRNAs of Goat Submandibular Glands Among Three Developmental Stages Are Involved in Immune Functions
Source: Front Genet. 2021 Jun 15;12:678194. doi: 10.3389/fgene.2021.678194 (PMC8239366; doi:10.3389/fgene.2021.678194)
Supplement: Supplementary Table 1 — The qRT-PCR primers used for validation. [file Table_1.DOCX]

Table S1 The qRT-PCR primers used for validation

| **ID** | **primer sequence** | **Base number** |
| --- | --- | --- |
| chi-miR-191-5p | CAACGGAATCCCAAAAGCAGCT | 22 |
| chi-miR-141 | TAACACTGTCTGGTAAAGATGG | 22 |
| chi-miR-16a-5p | TAGCAGCACGTAAATATTGGAG | 22 |
| chi-miR-128-3p | TCACAGTGAACCGGTCTCTTT | 21 |
| chi-miR-342-3p | TCTCACACAGAAATCGCACCCA | 22 |
| chi-miR-92a-3p | TATTGCACTTGTCCCGGCCTGT | 22 |
| chi-miR-363-3p | AATTGCACGGTATCCATCTGCG | 22 |
| chi-miR-29b-3p | TAGCACCATTTGAAATCAGT | 20 |
| chi-miR-193b-3p | AACTGGCCCACAAAGTCCCGCT | 22 |
| chi-miR-221-3p | AGCTACATTGTCTGCTGGGTTT | 22 |
| chi-miR-335-3p | TTTTTCATTATTGCTCCTGACC | 22 |
| chi-miR-20b | CAAAGTGCTCACAGTGCAGGTAG | 23 |
| miR-181-x | AACATTCAACGCTGTCGGTGAGT | 23 |
| miR-142-x | CATAAAGTAGAAAGCACTACT | 21 |
| miR-375-y | TTTGTTCGTTCGGCTCGCGTGA | 22 |
| miR-101-y | TACAGTACTGTGATAACTGACT | 22 |
| miR-142-y | TGTAGTGTTTCCTACTTTATGG | 22 |
| miR-181-y | ACCATCGACCGTTGATTGTACC | 22 |
| miR-106-y | CCGCACTGTGGGTACTTGCTGT | 22 |
| miR-3969-x | CCCATAAAGTAGAAAGCACTATA | 23 |
