# Supplementary material for: Differentially Expressed MiRNAs of Goat Submandibular Glands Among Three Developmental Stages Are Involved in Immune Functions
Source: Front Genet. 2021 Jun 15;12:678194. doi: 10.3389/fgene.2021.678194 (PMC8239366; doi:10.3389/fgene.2021.678194)
Supplement: Supplementary Table 2 — Results of miRNA alignment. [file Table_2.DOCX]

Table S2 Results of miRNA alignment

| Sample | Clean reads | Goat known miRNA | Goat known miRNA edit | Other known miRNA | Novel miRNA |
| --- | --- | --- | --- | --- | --- |
| A1-S | 12,076,850 (100.00%) | 8,127,543 (67.30%) | 1,158,815 (9.60%) | 1,926,087 (15.95%) | 8,998 (0.07%) |
| A2-S | 16,277,147 (100.00%) | 8,571,409 (52.66%) | 1,942,586 (11.93%) | 4,372,128 (26.86%) | 15,813 (0.10%) |
| A3-S | 13,361,013 (100.00%) | 9,208,321 (68.92%) | 1,290,429 (9.66%) | 2,078,164 (15.55%) | 10,639 (0.08%) |
| B3-S | 9,871,924 (100.00%) | 6,854,198 (69.43%) | 956,020 (9.68%) | 1,329,372 (13.47%) | 4,581 (0.05%) |
| B4-S | 11,821,567 (100.00%) | 7,045,349 (59.60%) | 1,491,169 (12.61%) | 1,746,303 (14.77%) | 7,612 (0.06%) |
| B5-S | 15,048,896 (100.00%) | 9,925,358 (65.95%) | 1,488,004 (9.89%) | 1,993,529 (13.25%) | 8,418 (0.06%) |
| C2-S | 13,723,498 (100.00%) | 8,814,600 (64.23%) | 1,265,619 (9.22%) | 1,880,710 (13.70%) | 8,077 (0.06%) |
| C3-S | 11,708,058 (100.00%) | 7,447,887 (63.61%) | 1,588,789 (13.57%) | 1,768,284 (15.10%) | 6,003 (0.05%) |
| C5-S | 11,938,948 (100.00%) | 8,057,984 (67.49%) | 1,687,010 (14.13%) | 1,613,895 (13.52%) | 4,982 (0.04%) |
