# Supplementary material for: Differentially Expressed MiRNAs of Goat Submandibular Glands Among Three Developmental Stages Are Involved in Immune Functions
Source: Front Genet. 2021 Jun 15;12:678194. doi: 10.3389/fgene.2021.678194 (PMC8239366; doi:10.3389/fgene.2021.678194)
Supplement: Supplementary Table 3 — Top three terms of Biological Process enrichment analysis of miRNA target genes with different expression patterns. [file Table_3.DOCX]

Table S3 Top three terms of Biological Process enrichment analysis of miRNA target genes with different expression patterns

|  | GO ID | Description | p.adjust |
| --- | --- | --- | --- |
| cluster 1 | GO:0010468 | regulation of gene expression | 5.40E-09 |
|  | GO:0006139 | nucleobase-containing compound metabolic process | 4.09E-08 |
|  | GO:0009059 | macromolecule biosynthetic process | 4.09E-08 |
| cluster 2 | GO:0006139 | nucleobase-containing compound metabolic process | 7.17E-08 |
|  | GO:0090304 | nucleic acid metabolic process | 2.33E-07 |
|  | GO:0046483 | heterocycle metabolic process | 3.00E-07 |
| cluster 3 | GO:0001932 | regulation of protein phosphorylation | 3.48E-05 |
|  | GO:0042325 | regulation of phosphorylation | 3.48E-05 |
|  | GO:0065009 | regulation of molecular function | 4.69E-05 |
| cluster 4 | GO:0051179 | localization | 1.13E-06 |
|  | GO:0006810 | transport | 1.63E-06 |
|  | GO:0051234 | establishment of localization | 3.68E-06 |
| cluster 5 | GO:0036315 | cellular response to sterol | 0.0096 |
|  | GO:0097306 | cellular response to alcohol | 0.0096 |
|  | GO:0036314 | response to sterol | 0.0234 |
